# Phenotypic characterization of *Synechocystis* sp. PCC 6803 substrains reveals differences in sensitivity to abiotic stress

**DOI:** 10.1371/journal.pone.0189130

**Published:** 2017-12-07

**Authors:** Tomáš Zavřel, Petra Očenášová, Jan Červený

**Affiliations:** Department of Adaptive Biotechnologies, Global Change Research Institute CAS, Brno, Czech Republic; Pacific Northwest National Laboratory, UNITED STATES

## Abstract

*Synechocystis* sp. PCC 6803 is a widely used model cyanobacterium, whose substrains can vary on both genotype and phenotype levels. Previously described phenotypic variations include ability of mixotrophic growth, ability of movement on agar plates and variations in pigments composition or cell size. In this study, we report for the first time significant variation among *Synechocystis* substrains in complex cellular traits such as growth rate, photosynthesis efficiency, cellular dry weight and cellular composition (including protein or carbohydrates content). We also confirmed previously reported differences in cell size. *Synechocystis* cultures were cultivated in controlled environment of flat panel photobioreactors under red, blue and white light of intensities up to 790 μmol(photons) m^-2^ s^-1^, temperatures 23°C–60°C, input CO_2_ concentrations ranging from 400 to 15 000 ppm and in BG11 cultivation medium with and without addition of NaCl. Three *Synechocystis* substrains were used for the comparative experiments: GT-L, GT-B (Brno, CZ) and PCC-B (Brno, CZ). Growth rates of *Synechocystis* GT-B were inhibited under high intensities of red light (585–670 nm), and growth rates of both substrains GT-B and PCC-B were inhibited under photons of wavelengths 485–585 nm and 670–700 nm. *Synechocystis* GT-B was more sensitive to low temperatures than the other two tested substrains, and *Synechocystis* GT-L was sensitive to the presence of NaCl in the cultivation media. The results suggest that stress sensitivity of commonly used *Synechocystis* substrains can strongly vary, similarly as glucose tolerance or motility as reported previously. Our study further supports the previous statement that emphasizes importance of proper *Synechocystis* substrains selection and awareness of phenotypical differences among *Synechocystis* substrains which is crucial for comparative and reproducible research. This is highly relevant for studies related to stress physiology and development of sustainable biotechnological applications.

## Introduction

Cyanobacteria, autotrophic prokaryotes, are widely used as model organisms for studying basic principles of photosynthesis, stress responses or evolutionary processes. They also possess great promises for utilization in biotechnology, mostly for their capacity to generate biomass on short timescale and to produce wide range of valuable compounds [1,2]. Among cyanobacteria, single unicellular *Synechocystis* sp. PCC 6803 (*Synechocystis* hereafter) gained a unique position since it was the first cyanobacterium with the entire genome sequenced [3,4]. *Synechocystis* is naturally and easily transformable by exogenous DNA [5] which, together with its stable and fast growth, makes this strain attractive for research in many laboratories worldwide. According to the database http://apps.webofknowledge.com (search for “Synechocystis 6803”), currently more than 4 400 research studies from altogether more than 1 300 laboratories refer to this strain.

The original *Synechocystis* strain (“Berkeley”) was isolated in Oakland, California in 1968 by R. Kunisawa [6] and it was deposited in Pasteur Culture Collection (strain PCC 6803) and in American Type Culture Collection (strain ATCC 27184). From these culture collections *Synechocystis* spread further, for more detailed history overview see [7]. Despite the common origin, it was reported that *Synechocystis* substrains in different laboratories (i.e. with different deposition and/or maintenance history) can vary on both genotype and phenotype levels. Several *Synechocystis* substrains were re-sequenced in addition to the first sequenced Kazusa strain [3,4] and several specific (mostly single-point) mutations were identified within genes related to photosynthesis, transport, or motility [7–13]. In addition, variability in genome copy number was reported recently [14]. However, even though the documented phenotypic variations include changes in essential cellular parameters such as content and composition of photosynthetic complexes [12], pigment composition [12,15], cell size [15], motility [7,13] or glucose tolerance [7,16], variations among *Synechocystis* substrains in the complex cellular traits such as growth rates were never reported up to date.

**Fig 1:**
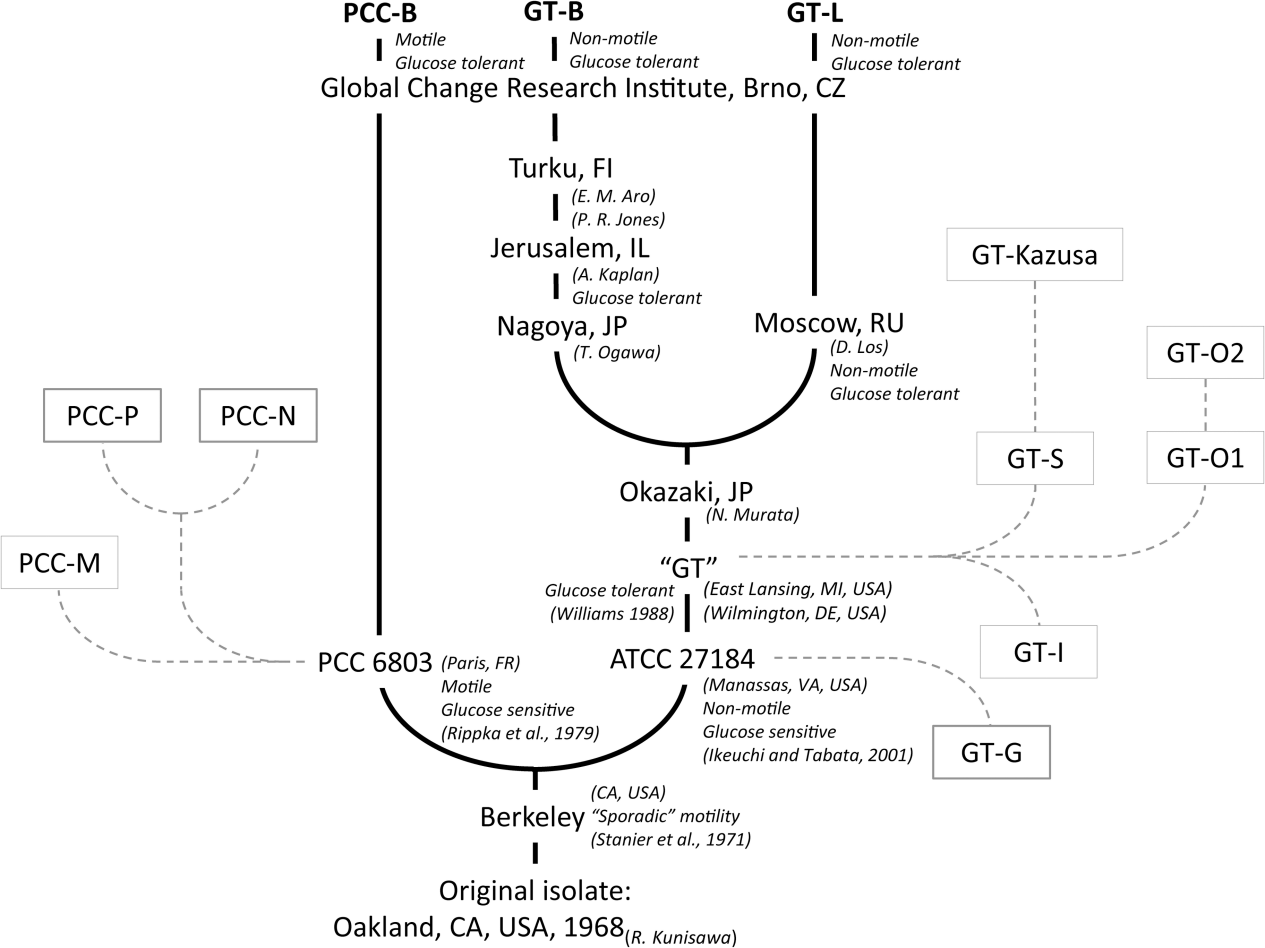
History of the *Synechocystis* sp. PCC 6803 substrains PCC-B, GT-B and GT-L as used in the current study (full lines), and their putative relation to *Synechocystis* substrains that have identified genome sequences (dashed lines, according to Ding et al. (2015) [13]). The phenotypic properties of individual *Synechocystis* substrains are listed in accordance with Ikeuchi and Tabata (2001), Rippka et al. (1979), and Williams (1988) [7,16,50], as well as in accordance to personal communication with members of laboratories in Jerusalem and in Moscow.

The main aim of this work was to investigate phenotypic response of *Synechocystis* substrains with different deposition history to a wide range of environmental conditions. The specific objectives were to identify possible differences among the tested *Synechocystis* substrains in: 1) growth rates, 2) photosynthesis performance and 3) biochemical composition of *Synechocystis* cells during cultivation under optimal and suboptimal conditions. The cultures were growing in a controlled environment of flat panel photobioreactors [17–19]. In addition to the “standard” (modest) cultivation conditions, *Synechocystis* was cultivated under high intensities of red light (up to 660 μmol(photons) m^-2^ s^-1^), blue light (up to 220 μmol(photons) m^-2^ s^-1^) and white light (up to 790 μmol(photons) m^-2^ s^-1^), in a wide range of temperatures (23°C–60°C) as well as with varying salinity of the cultivation media (0 and 0.5 mM NaCl). Three *Synechocystis* substrains were used for the comparative growth experiments: two substrains were derived from the “ATCC” lineage and one substrain was derived from the “PCC” lineage. *Synechocystis* derived from the “ATCC” lineage included substrain GT-L (described previously in Zavřel et al. (2015) [19]) and newly denominated substrain GT-B (according to Brno, CZ). The substrain derived directly from Pasteur Culture Collection was denominated as PCC-B (similarly, according to Brno, CZ). For details on the substrains history, see Chapter 2.1 and ***Fig 1***.

Throughout the experiments, strong and complex phenotypic variation among the *Synechocystis* substrains was observed in a wide range of cultivation conditions. The variability included changes in growth rates, photosynthesis efficiency and cellular composition (including proteins or carbohydrates content, dry weight or cell size). Additionally, each *Synechocystis* substrain was found to be sensitive or tolerant to different environmental stress factor. The substrain GT-L was sensitive to the presence of salt in the cultivation media, whereas the substrains GT-B and PCC-B were sensitive to high intensities of red light and surprisingly also to low amounts of light of wavelengths 485–585 nm and 670–700 nm. Additionally, the substrain GT-B was more sensitive to low temperatures than the other two tested substrains. Under standard laboratory conditions (low light, growth temperature close to optimum, standard cultivation media), only minor variations in the tested phenotypic parameters were observed- which is consistent with the previous reports [15].

The results of this study bring new insight into our understanding to the complexity of phenotypic variability in the model cyanobacterium *Synechocystis* sp. PCC 6803. The results emphasize importance to consider specific cultivation requirements and capabilities of stress tolerance of each selected *Synechocystis* substrain. This can be expected to represent a crucial aspect in studies that address effects of environmental stress on *Synechocystis* physiology. Additionally, our results can have large consequences for biotechnological applications that consider *Synechocystis* as a production strain.

## Material and methods

### History of the strains

Three *Synechocystis* substrains were used for the comparative growth experiments. Substrain GT-L was kindly provided by Prof. D. A. Los (Timiryazev Institute of Plant Physiology, Russian Academy of Sciences, Moscow, RU) in 2013. Growth capacities of this strain were described in detail previously [19]. To compare phenotypic signature of this substrain with another glucose tolerant *Synechocystis*, a substrain kindly provided by Dr. Patrik R. Jones was used (Imperial College London, GB, when providing the strain (early 2012), Dr. P. R. Jones was staying at the University of Turku, FI). This substrain was designated as GT-B (Brno, CZ). As a reference, *Synechocystis* sp. PCC 6803 regularly ordered from Pasteur Culture Collection (Paris, FR) in late 2013 was used, denoted as PCC-B (Brno, CZ). Full history of all three substrains used in this work represented in ***Fig 1***.

After receiving, all substrains were resuspended in fresh cultivation medium BG11 [6] supplemented with 17 mM HEPES (Carl Roth, Karsruhe, Germany) and cultivated on standard orbital shaker in the cultivation chamber as described in the next section. After reaching late exponential / early linear phase, the cells were gently centrifuged (2000 g, 10 min, 25°C), resuspended in 10 ml of fresh cultivation medium, supplemented with methanol to a final concentration of 5%, and long-term cryopreserved in -80°C until further work.

### Inoculum cultures conditioning

Before comparative growth experiments, the frozen cultures were thawed on ice in the dark. After thawing, the cultures were resuspended in fresh BG11 medium (supplemented with 17 mM HEPES) in 250 ml Erlenmeyer flask, placed on orbital shakers (120 rpm) and covered with a paper tissue for at least 24 hours to protect the cells from the excess of light. The inoculum cultures were cultivated on orbital shakers in a cultivation chamber that was tempered at 31°C under illumination of intensity 110 μmol(photons) m^-2^ s^-1^ (provided by warm white light LEDs) and under ambient air atmosphere. Each strain was maintained on orbital shakers for a period of one to three months; during that time all cultures were periodically resuspended in a fresh cultivation medium at least twice a month.

All cultures were periodically checked for contamination using standard LB agar plates. In addition, the cultures were routinely checked for contamination prior to their inoculation into the photobioreactors, either using standard LB agar plates or using ImageStream X MkII imaging flow cytometer (Amnis Corp., Seattle, WA, USA). The cytometric detection of bacterial contamination was based on SYBR^®^ Green I nucleic acids gel staining (Thermo Fisher Scientific, Waltham, MA USA) that marks DNA of both cyanobacteria and bacteria cells. Nucleic acids were marked by addition of 5 μl of SYBR^®^ Green I solution (diluted 1:100 in DMSO) to 500 μl of moderate dense cells suspension. The samples were incubated for 10 min in dark at laboratory temperature. During the cytometric analysis, 488 nm argon laser was used to excite both SYBR^®^ Green I and chlorophyll *a*, and 642 nm laser was used to excite phycobilisomes. To identify *Synechocystis* cells within all measured objects, gating of the measured populations was applied to discriminate: a) focused objects, b) objects with both pigments as well as with SYBR^®^ Green fluorescence, c) single cell and round objects (width/ length ratio between 0.8–1.0) and d) objects of reasonable size (2–5 μm in diameter). The bacteria cells were discriminated as following: a) focused objects, b) objects with SYBR^®^ Green fluorescence and no chlorophyll fluorescence, and c) object smaller than 2 μm in diameter.

For comparative growth experiments, only inoculum cultures with no bacteria proliferation as observed with the use of the standard LB agar plates were used. In addition, both inoculum and running cultures in the photobioreactors that contained more than 2% of bacteria (as detected by ImageStream MkII imaging flow cytometer) were always discarded and not used for any further work. Contamination in the selected range (0–2% bacteria) did not have any impact on the growth rates or any other measured parameter–within the selected contamination range, the results were always highly reproducible for all *Synechocystis* substrains.

### Photobioreactor

Comparative growth experiments were performed in flat panel photobioreactors that were described in detail previously [17]. Two types of photobioreactor illumination panels were used. The first panel was designed as a chessboard configuration of red and blue LEDs, and the second panel was designed as a chessboard configuration of red and white LEDs, respectively (red: λmax ≈ 633 nm, λ1/2 ≈ 20 nm, Luxeon LXHLPD09; blue: λmax ≈ 445 nm, λ1/2 ≈ 20 nm, Luxeon LXHL-PR09; white: Luxeon LXHL-PW09; all manufactured by Future Lighting Solutions, Montreal, QC, Canada). Spectral characteristics of the LEDs are shown in [19]. The photobioreactor continuously measured optical density (OD) by an inbuilt densitometer and steady-state pigment fluorescence emission yield by a build-in fluorometer (both described in [17]). Dissolved O_2_ was monitored by InPro6800 electrodes (Mettler-Toledo, Inc., Columbus, OH, USA). Culture homogenization (mixing) was secured by inflow gas bubbling with rate of 200 ml min^-1^. The bubbling was complemented by rotation of magnetic stirrer bar (ø5 × 35 mm, 210 rpm) in a vertical plane. Culture temperature and pH were monitored by InPro3253 electrode (MettlerToledo, Inc.). All other photobioreactor accessories were the same as described in [19].

### Experimental setup

The growth characterization was performed in two regimes: in a quasi-continuous regime and in a batch regime. The quasi-continuous cultivation setup (turbidostat) was operated in accordance with Zavřel et al. (2015) [19]. Briefly, the exponentially growing cells were maintained in a defined optical density range (measured at 680 nm, OD_680_) by controlled dilution by fresh cultivation medium. The optical density was measured by the photobioreactor optical detector, and the OD_680_ range was set to 0.52–0.58, which corresponded to approximately 2–4 x 10^7^ cells ml^-1^ (Fig A in S1 File). Starting OD_680_ of all cultures was 0.1–0.2, which corresponded to approximately 2–4 x 10^6^ cells ml^-1^. Once the culture density reached OD_680_ 0.58, the quasi-continuous cultivation setup was initiated by starting automated culture dilution within the selected OD_680_ range. During the quasi-continuous regime, the cultures were growing under each specific cultivation condition for at least 24 hours. This period was long enough to reach growth stability, i.e. to acclimate the cells to specific cultivation conditions. Growth rates were calculated from dynamics of OD_680_ increase in the specified OD_680_ range. Growth stability was evaluated by measuring at least three reproducible successive growth rates, as calculated from independent growth periods. At 680 nm, light is absorbed by both cells structure (e.g. cell count and morphology) and pigments content (mostly chlorophyll *a* in the case of *Synechocystis*). The use of OD_680_ for growth evaluation [19] allowed us to detect stabilization of both combined cells characteristics. Moreover, in the quasi-continuous characterization setup, biochemical composition of the cells (including pigment content) kept stable for each examined condition after growth stabilization and thus OD_680_ readouts could be reliably used for growth rate determination with more favorable signal-to-noise ratio when compared to OD_720_. In the course of quasi-continuous experiments, the tested *Synechocystis* substrains were cultivated under red light of intensities 25–660 μmol(photons) m^-2^ s^-1^, blue light of intensities 0–220 μmol(photons) m^-2^ s^-1^, and white light of intensities 0–790 μmol(photons) m^-2^ s^-1^, and in the temperature range 23°C–38°C.

**Fig 2:**
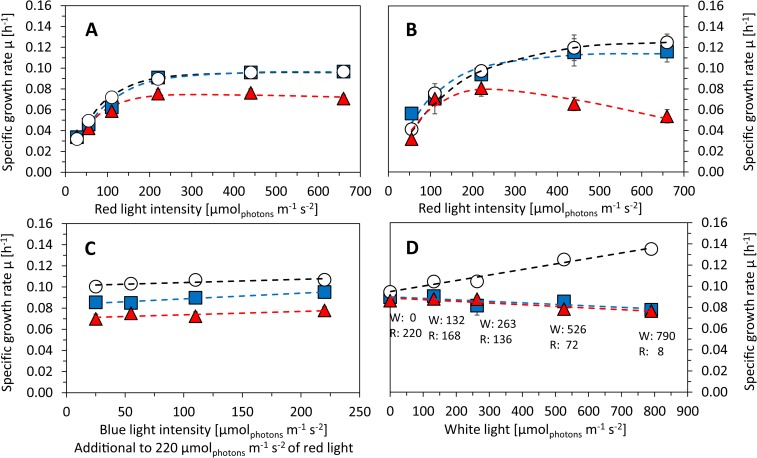
Growth rates of *Synechocystis* sp. PCC 6803 substrains GT-L (white circles), GT-B (red triangles) and PCC-B (blue squares) under increasing red, blue and white light. A, B–Red light was set to intensities of 25–660 μmol(photons) m^-2^ s^-1^ and it was supplemented with 25 μmol(photons) m^-2^ s^-1^ of blue light. The experiments were performed at 32°C (A) and 35°C (B). C–Red light of 220 μmol(photons) m^-2^ s^-1^ was supplemented with 25–220 μmol(photons) m^-2^ s^-1^ of blue light under 32°C. D–Red light of 220–8 μmol(photons) m^-2^ s^-1^ was supplemented with white light of 0–790 μmol(photons) m^-2^ s^-1^ (at 32°C) in order to keep the red photons (as a combination of red light and part of the white spectra) constant at 220 μmol(photons) m^-2^ s^-1^ (according to [19]). All experiments were carried out in a quasi-continuous regime as described in the main text under growth saturating CO_2_ concentration of 5 000 ppm. Each point represents average of at least four independent experiments, error bars represent standard errors. The dashed lines in panels A and B represent fitting of the data points by the function derived by Platt et al. (1980) [51]. The dashed lines in panels C and D represent linear fitting of the data points by the least squares method. Legend in panel D represents particular combinations of white light (W) and red light (R) in units of μmol(photons) m^-2^ s^-1^.g.

Under higher temperatures, the cultures were not able to grow in the quasi-continuous regime any more, and it was only possible to introduce the heat shock for short time during a batch cultivation. The batch regime was operated according to [20]. The batch conditions were set to be saturating for growth of all tested *Synechocystis* substrains from at least 95%, i.e. 220 μmol(photons) m^-2^ s^-1^ of red light, 25 μmol(photons) m^-2^ s^-1^ of blue light, and 32°C (see ***Fig 2*** and Results for further details). *Synechocystis* sensitivity to NaCl was tested in the batch regime under the same cultivation conditions. During all batch experiments, *Synechocystis* was supplemented with CO_2_ concentration of 5 000 ppm, which was saturating for growth of all tested substrains (Fig B in S1 File).

### Analytical methods

The oxygen evolution/respiration rates (photosynthetic-irradiance curves) were measured within the photobioreactor cuvette by turning off aeration for 10 min, during a 5 min dark period followed by a 5 min light period, according to [20]. Pigment fluorescence curves (O-J-I-P-S-M) were measured by Fluorometer FL-100 (Photon System Instruments Ltd., Brno, CZ) which was equipped with 635 nm LED of intensity c. 2 000 *μ*mol(photons) m^-2^ s^-1^ for pigments fluorescent excitation (the LED provided both actinic and measurement light). Carbon uptake was evaluated using Gas Analyzing System (Photon System Instruments Ltd., Brno, CZ), described in detail previously [18]. The mixture of air and CO_2_ was bubbled for 5 min through the photobioreactor cuvette in a closed loop, where the only carbon loss was possible due to the photosynthetic fixation.

Content of chlorophyll *a* and total carotenoids was measured spectrophotometrically following protocol of Zavřel et al. (2015) [21]. Content of phycobilisomes was measured by modified method of Bennett et Bogorad (1973) [22]. Briefly, 10 ml of culture suspension was collected in 15-ml falcon tubes and centrifuged (4 000 g, 10 min, 4°C), supernatant was discarded and pellet was stored in -80°C for 2 months until further processing. After thawing on ice, the pellets were resuspended in PBS buffer (pH 7.4) and centrifuged (4 000 g, 10 min, 4°C). Supernatant was partially discarded and the cells were transferred to Eppendorf tubes on ice. The Eppendorf tubes were centrifuged again (15 000 g, 10 min, 4°C), supernatant was discarded and the tubes were put to -80°C for 30 min. Frozen samples were lyophilized overnight. After lyophilization, glass beads were added to dry cellular pellets and the samples were homogenized on Silamat S6 homogenizer (Ivoclar Vivadent AG, Schann, LI) for 30 sec. After homogenization, PBS buffer (4°C) was added to the tubes for the phycobilisomes extraction, the samples were vortexed for 3 sec and put on ice in the dark for 30 min. After extraction, the samples were centrifuged (15 000 g, 10 min and 4°C) and concentration of phycobilisomes was measured with the use of spectrophotometer UV 2600 (Shimadzu Scientific Instruments, Inc., Columbia, MD, USA) according to following equations:

$$\mathrm{Phycocyanin}=\left(A_{615}-0.474*A_{652}\right)/5.34\;\left[\mathrm{mg}\;{\mathrm{ml}}^{\text{-}1}\right]$$

$$\mathrm{Allophycocyanin}=\left(A_{652}-0.208*A_{615}\right)/5.09\;\left[\mathrm{mg}\;{\mathrm{ml}}^{\text{-}1}\right]$$

Content of cellular proteins was measured by Bicinchoninic Acid Protein Assay Kit (Sigma-Aldrich, St. Louis, MO, USA), using bovine serum albumin as a standard, and following instructions of the manufacturer. Content of cellular saccharides was measured according to [23]. Briefly, after harvesting, 1 ml of cellular suspension was centrifuged (20 000 g, 15 min, 25°C) and the pellet was resuspended in 1 ml of distilled water. 8 x 30 *μ*l of each sample was transferred to 96-well plate, and 30 *μ*l of 5% phenol, followed by 150 *μ*l of 96% H_2_SO_4_ was added to each well (after each step, the suspension was mixed by gentle pipetting). The plates were covered with the original plate lid and the samples were incubated at laboratory temperature for 60 min. The concentration of cellular saccharides was measured by Multiskan™ GO Microplate Spectrophotometer (Thermo Fisher Scientific, Waltham, MA, USA) at 490 nm, using D-glucose as a standard.

Content of glycogen was measured according to [20]. After harvesting from the photobioreactor, 10 ml of culture suspension was centrifuged (4 000 g, 15 min), supernatant was discarded and pellet was resuspended in 5 ml of distilled water. Aliquots of 480 *μ*l were transferred to eight Eppendorf tubes. The samples were centrifuged (20 000 g, 10 min, 4°C), supernatant was discarded and 480 *μ*l of cold methanol was added to each tube. The samples were homogenized by gentle pipetting, placed to +4°C for 20 min and centrifuged again at 4°C. The methanol supernatants were used for analysis of chlorophyll *a* and carotenoids as described above and the pellets were stored in -80°C until further processing for up to 3 months. After thawing on ice, the pellets were resuspended in 200 *μ*l of 30% KOH and they were incubated at 95°C for 90 min. After cooling down at laboratory temperature, the samples were mixed with 1.2 ml of cold ethanol (+4°C) and stored at -20°C overnight. The precipitated carbohydrates were then separated by intensive centrifugation (20 000 g, 70 min, 4°C). The supernatant was discarded and the pellet was dried at 60°C for 30 min in Vacuum Concentrator 5305 (Eppendorf, Hamburg, DE). Dry pellets were diluted in 480 *μ*l of deionized water and transferred to 96-well plate. Content of carbohydrates in the samples was measured spectrophotometrically according to [23], following the same procedure as for determination of total cellular saccharides.

Cell size was determined using ImageStream MkII imaging flow cytometer (Amnis Corp., Seattle, WA, USA). Right after harvesting from the photobioreactor, 500 *μ*l of the culture suspension was centrifuged (4 000 g, 4 min, 25°C), supernatant was discarded, pellet was resuspended in 0.25% glutaraldehyde solution and the samples were incubated for 10 min at laboratory temperature. The fixed cells were stored in -80°C until further processing (up to 1 month in total). For further analysis, the samples were thawed on ice for 2 hours, and they were kept at laboratory temperature for additional 30 min after thawing. During the cytometric analysis, only bright field images were collected. Gating of the measured populations was applied to discriminate: a) focused objects (using combination of both RMS gradient and Treshold Mask features of IDEAS® software), and b) round objects (width/ length ratio between 0.9–1.0). The imaging flow cytometer was calibrated with nonfluorescent microspheres (1–15 *μ*m, Thermo Fisher Scientific, Waltham MA, USA) and the results were validated with light microscope Axio Imager 2 (Carl Zeiss, Oberkochen, DE). Cellular dry weight was measured using XA105DR analytical balances (Mettler Tolledo, Greifensee, CH). Cell count was measured with Cellometer Auto M10 (Nexcelom Bioscience, Lawrence, MA, USA).

To test statistical differences among *Synechocystis* substrains under the tested conditions, one-way ANOVA followed by Tukey’s HSD post-hoc test was applied, using STATISTICA ® software (Dell Inc., Tulsa, OK, USA).

## Results

### General features of the tested *Synechocystis* substrains

All *Synechocystis* substrains used in this study were tolerant to the presence of 5 mM glucose in liquid cultivation medium as well as in solid BG11 agar plates. *Synechocystis* substrains GT-B and GT-L were not motile, whereas substrain PCC-B showed negative phototaxis on agar plates (top illumination by warm white light through a 3 cm circular area in the middle of standard 10 cm agar plate). This is in agreement with previously published motility distribution among PCC-derived and ATCC-derived *Synechocystis* substrains [10,13]. All *Synechocystis* substrains used in this study were not sensitive to the presence of shear stress introduced by rotation of the cylindrical stirrer bar inside the cuvette up to 210 rpm or maximal circular velocity 0.4 m s^-1^. Also intensity of bubbling between 50–200 ml min^-1^ had no effect on the growth of all three tested Synechocystis substrains (tested with CO_2_ input concentration of 5 000 ppm, Fig C in S1 File).

### *Synechocystis* sensitivity to high light

#### Red light (585–670 nm)

During quasi-continuous cultivation under 25–660 *μ*mol(photons) m^-2^ s^-1^ of red light, the cultures were supplemented with 25 *μ*mol(photons) m^-2^ s^-1^ of blue light (***Fig 2A and 2B***), following the same setup as introduced previously [19]. The red light sensitivity experiments were performed at 32°C (temperature that saturated growth of substrain GT-L from 95%), and at 35°C (optimal growth temperature for this substrain [19]). Growth rates of both substrains GT-L and GT—B were not photoinhibited even under 660 *μ*mol(photons) m^-2^ s^-1^ of red light. However, growth rates of substrain GT-B started to decrease under red light of intensities higher than 220 *μ*mol(photons) m^-2^ s^-1^ (***Fig 2A***). This growth was even more pronounced when the cultivation temperature was increased from 32°C to 35°C (***Fig 2B***).

**Fig 3:**
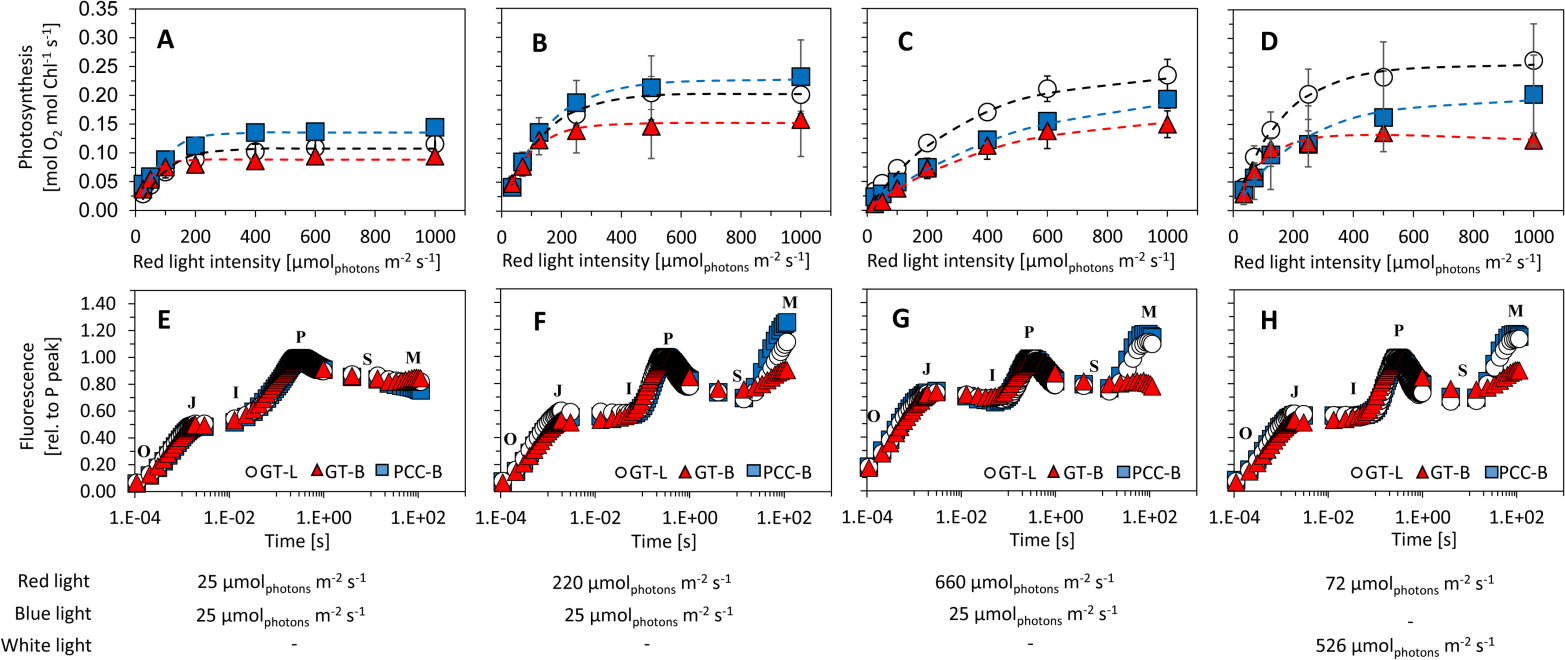
**Photosynthesis performance of *Synechocystis* substrains GT-L (white circles), GT-B (red triangles) and PCC-B (blue squares) evaluated by measurement of oxygen evolution (A, B, C, D) and fast pigment fluorescence kinetics (O-J-I-P-S-M; E, F, G, H)**. The cultures were adapted to 25 μmol(photons) m^-2^ s^-1^ of red and blue light (A, E), 220 μmol_photons_ m^-2^ s^-1^ of red light complemented with 25 μmol(photons) m^-2^ s^-1^ of blue light (B, F), 660 μmol(photons) m^-2^ s^-1^ of red complemented with 25 μmol(photons) m^-2^ s^-1^ of blue light (C, G) and 72 μmol_photons_ m^-2^ s^-1^ of red light complemented with 526 μmol(photons) m^-2^ s^-1^ of white light (D, H). The cells were cultivated at 32°C under input CO_2_ concentration of 5 000 ppm in a quasi-continuous regime as described in the main text. The cells were darkened for 15 minutes prior to fluorescence measurement. Each point represents average from at least four independent experiments, the error bars represent standard errors. The pigment fluorescence curves are visualized without error bars for better clarity. The dashed lines in panels A–D represent fitting of the data points by the function derived by Platt et al. (1980) [51].

To understand the increased sensitivity of substrain GT-B to high red light, additional set of measurements was performed that aimed on estimation of *Synechocystis* photosynthesis capacity and efficiency. Photosynthesis-irradiance curves (P-I curves) of all three *Synechocystis* substrains under low (25 *μ*mol(photons) m^-2^ s^-1^) and high red light (220 and 660 *μ*mol(photons) m^-2^ s^-1^) are presented on ***Fig 3A–3C***, together with P-I curves measured under high white light (***Fig 3D***). Derived parameters α (initial slope of the P-I curves, reflecting photosynthesis efficiency under low irradiances) and Pmax (maximal photosynthetic capacity) are summarized in Table A in S1 File. Under low light (25 *μ*mol(photons) m^-2^ s^-1^ of both red and blue light), all tested *Synechocystis* substrains showed lower Pmax (0.09–0.14 mol(O_2_) mol(Chl^-1^) s^-1^) than under increased irradiances. Under high red light (220 *μ*mol(photons) m^-2^ s^-1^ and 660 *μ*mol(photons) m^-2^ s^-1^), substrain GT-B showed the lowest photosynthetic capacity (0.14 and 0.18 mol(O_2_) mol(Chl^-1^) s^-1^, respectively) when compared to substrains GT-L (0.21 and 0.24 mol(O_2_) mol(Chl^-1^) s^-1^, respectively) and PCC-B (0.23 and 0.21 mol(O_2_) mol(Chl^-1^) s^-1^, respectively). Substrain GT-B had also slightly reduced carbon fixation efficiency under 220 *μ*mol(photons) m^-2^ s^-1^ of red light (0.15±0.03 fg(CO_2_) cell^-1^ s^-1^) when compared to substrains GT-L (0.24±0.07 fg(CO_2_) cell^-1^ s^-1^) and PCC-B (0.2±0.02 fg(CO_2_) cell^-1^ s^-1^, Fig D in S1 File), however, the differences in carbon fixation were not statistically significant (ANOVA: p>0.05).

To provide additional insight into operation of photosynthetic electron transport chain, a series of fluorescence induction curves (O-J-I-P-S-M) was measured under low and high red light (***Fig 3E–3G***) as well as under high white light (***Fig 3H***). Initial part of the fluorescence induction curve (O-J-I-P, the time frame <1 sec) was almost identical for all *Synechocystis* substrains, which was confirmed also by observing minimal differences in derived O-J-I-P curve parameters (Fig E in S1 File). Also P-S fluorescence dip (c. 1–20 sec of illumination) was similar in all tested *Synechocystis* substrains. However, the amplitude of S-M fluorescence rise (c. 20 -120 sec of illumination) was significantly reduced under both high red and white light in substrain GT-B (***Fig 3***), which suggests differences in photosynthesis induction in this substrain.

**Fig 4:**
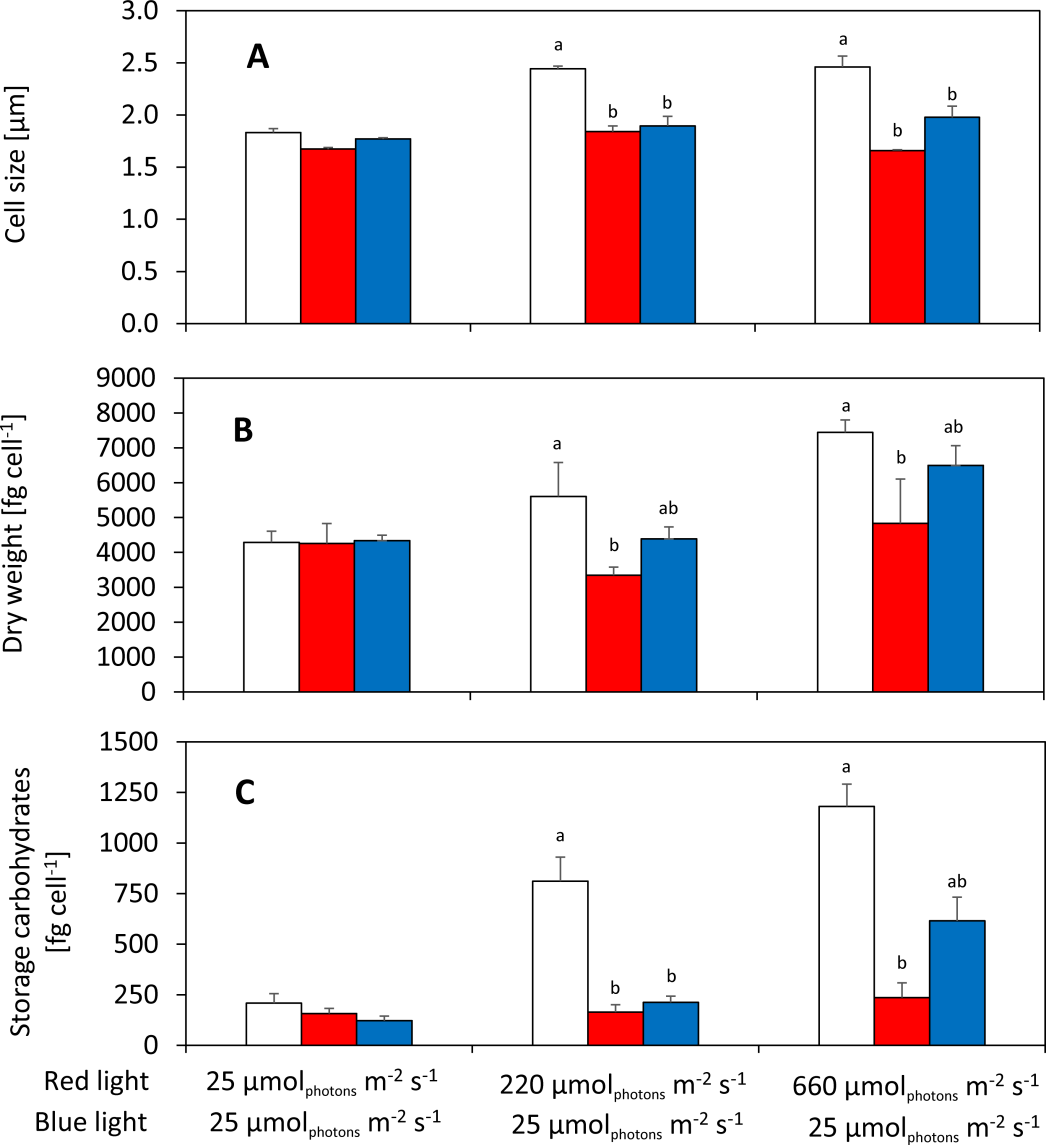
Cell size (A), dry weight (B), and glycogen content (C) of *Synechocystis* substrains GT-L (white bars), GT-B (red bars) and PCC-B (blue bars) under 25, 220 and 660 μmol(photons) m^-2^ s^-1^ of red light complemented with 25 μmol(photons) m^-2^ s^-1^ of blue light. The cells were cultivated at 32°C under input CO_2_ concentration of 5 000 ppm in a quasi-continuous regime as described in the main text. Each value represents average from at least three independent experiments, error bars represent standard errors. Differences in cell size, dry weight and glycogen content among *Synechocystis* substrains are marked by letters above the particular columns (Tukey’s HSD post-hoc test following one-way ANOVA: p<0.05).

Under high intensities of red light, content of cellular pigments (including chlorophyll *a*, total carotenoids, phycocyanin and allophycocyanin) was similar in the cells of all tested *Synechocystis* substrains (Fig F in S1 File). On the other hand, cellular composition of the tested *Synechocystis* substrains (including content of cellular carbohydrates, ***Fig 4***, and proteins, Fig G in S1 File) varied under high red light, similarly as cell size and cellular dry weight (***Fig 4***). The substrain GT-L was having cells of the largest size under 220 and 660 *μ*mol(photons) m^-2^ s^-1^ (c. 2.4 *μ*m), and on the contrary the substrains GT-B and PCC-B had smaller cells (1.7–2.0 *μ*m, ***Fig 4A***). Cellular dry weight under high red light (660 *μ*mol(photons) m^-2^ s^-1^) was the highest in substrain GT-L (7443±356 fg cell^-1^) and the lowest in substrain GT-B (4836±1267 fg cell^-1^, ***Fig 4B***). Cellular dry weight expressed as mg l^-1^ was not varying among *Synechocystis* substrains (Fig H in S1 File), following similar cellular concentration in all cultures (Fig A in S1 File). Content of intracellular polysaccharides (glycogen) under the same conditions was also the highest in substrain GT-L (1181±110 fg cell^-1^) and the lowest in substrain GT-B (236±72 fg cell^-1^, ***Fig 4C***). Similarly, content of total cellular carbohydrates under high red light was the lowest in substrain GT-B (802±168 fg cell^-1^) when compared to the other two substrains (1870–3011 fg cell^-1^, Fig I in S1 File). Protein content under low and high red light (25 and 660 *μ*mol(photons) m^-2^ s^-1^) was interestingly higher in substrain PCC-B (2516±147 fg cell^-1^) than in the other two substrains (1804–2178 fg cell^-1^, Fig G in S1 File).

The above mentioned results suggests that substrain GT-L was able to utilize red photons with the best efficiency. On the other hand, substrain GT-K was clearly photoinhibited under high intensities of red light, which also affected its ability to accumulate sugars or proteins.

#### Blue light (405–485 nm)

In order to test the effect of other than red wavelengths on *Synechocystis* growth, the three *Synechocystis* substrains were cultivated also under constant red light of 220 *μ*mol(photons) m^-2^ s^-1^ (light intensities saturating growth of all substrains from at least 95% at 32°C, ***Fig 2A***) with addition of blue light of intensities 25–220 *μ*mol(photons) m^-2^ s^-1^. As shown in ***Fig 2C***, none of the tested *Synechocystis* substrains were sensitive to blue light up to 220 *μ*mol(photons) m^-2^ s^-1^. On the contrary, growth of all tested *Synechocystis* substrains slightly increased after addition of blue photons to growth saturating red light.

### Photons of wavelengths 485–585 nm and 670–700 nm

Additionally, the three *Synechocystis* substrains were cultivated under high intensities of white light. White light spectrum (405–700 nm) contained 27% of red photons (585–670 nm), 18% of blue photons (405–485 nm) and 55% of photons of other wavelengths (485–585 nm and 670–700 nm). To test effect of non-red and non-blue photons on *Synechocystis* growth, the red photons (as a combination of the whole spectrum of red light and a portion of white light spectrum) were kept on the constant level of 220 *μ*mol(photons) m^-2^ s^-1^ (intensity saturating growth of all substrains from more than 95% at 32°C, ***Fig 2A***) by sequential stepwise increase of white light (up to 790 *μ*mol(photons) m^-2^ s^-1^) followed by simultaneous stepwise decrease of red light (up to 8 *μ*mol(photons) m^-2^ s^-1^). Particular red and white light combinations (as listed in ***Fig 2D***) were set such that the blue photons (as part of the white light spectrum) were increasing up to 150 *μ*mol(photons) m^-2^ s^-1^ and non-red and non-blue photons (485–585 nm and 670–700 nm) were increasing up to 435 *μ*mol(photons) m^-2^ s^-1^.

Growth of substrain GT-L was enhanced by photons of 485–585 nm and 670–700 nm, whereas growth of substrains GT-B and PCC-B was inhibited under these wavelengths (***Fig 2D***). We performed again a series of additional measurements to reveal nature of the white light sensitivity of substrains PCC-B and GT-B. The maximal photosynthetic capacity of both substrains PCC-B and GT-B slightly decreased under high white light (526 *μ*mol(photons) m^-2^ s^-1^ of white light complemented by 72 *μ*mol(photons) m^-2^ s^-1^ of red light, the white light was containing 290 *μ*mol(photons) m^-2^ s^-1^ of wavelengths 485–585 nm and 670–700 nm, ***Fig 3***). On the contrary, photosynthetic capacity of substrain GT-L slightly increased under high white light.

Interestingly, fluorescence induction (O-J-I-P-S-M) under high intensities of white light had almost identical trends as observed under high intensities of red light, i.e. the O-J-I-P transition was almost identical for all tested substrains and the S-M fluorescence rise was much less pronounced in the substrain GT-B. This result suggests distinct mechanisms of growth and photosynthesis inhibition under red photons (585–670 nm) and under non-red and non-blue photons (485–585 nm and 670–700 nm).

Content of cellular carbohydrates under high white light, (both total carbohydrates and glycogen) followed the same trends as under high red light—the lowest concentrations were measured in substrain GT-B (774±55 fg(total saccharides) cell^-1^ and 146±30 fg(glycogen) cell^-1^, respectively) when compared to the other two substrains (1368–2691 fg(total saccharides) cell^-1^ and 398–828 fg(glycogen) cell^-1^, respectively, Figs I and J in S1 File). Interestingly, *Synechocystis* substrain PCC-B was able to increase content of cellular saccharides under high intensities of white light even though its growth and photosynthesis were both inhibited under these conditions (Figs I and J in S1 File).

To test whether the photons of 485–585 nm and 670–700 nm had an instant photoinhibition effect, we cultivated substrain PCC-B under low intensities of white light. Addition of 56 *μ*mol(photons) m^-2^ s^-1^ of white light (which contained only 31 *μ*mol(photons) m^-2^ s^-1^ of 485–585 nm and 670–700 nm photons) already caused inhibition of growth rates of this substrain, and with stepwise increase of white light intensity, the photoinhibition effect was intensifying (Fig K in S1 File). The immediate growth inhibition of the substrain PCC-B under the photons of wavelengths 485–585 nm and 670–700 nm also suggests distinct photoinhibition mechanism under these photons and red photons.

### *Synechocystis* sensitivity to suboptimal temperature

#### Low temperature

**Fig 5:**
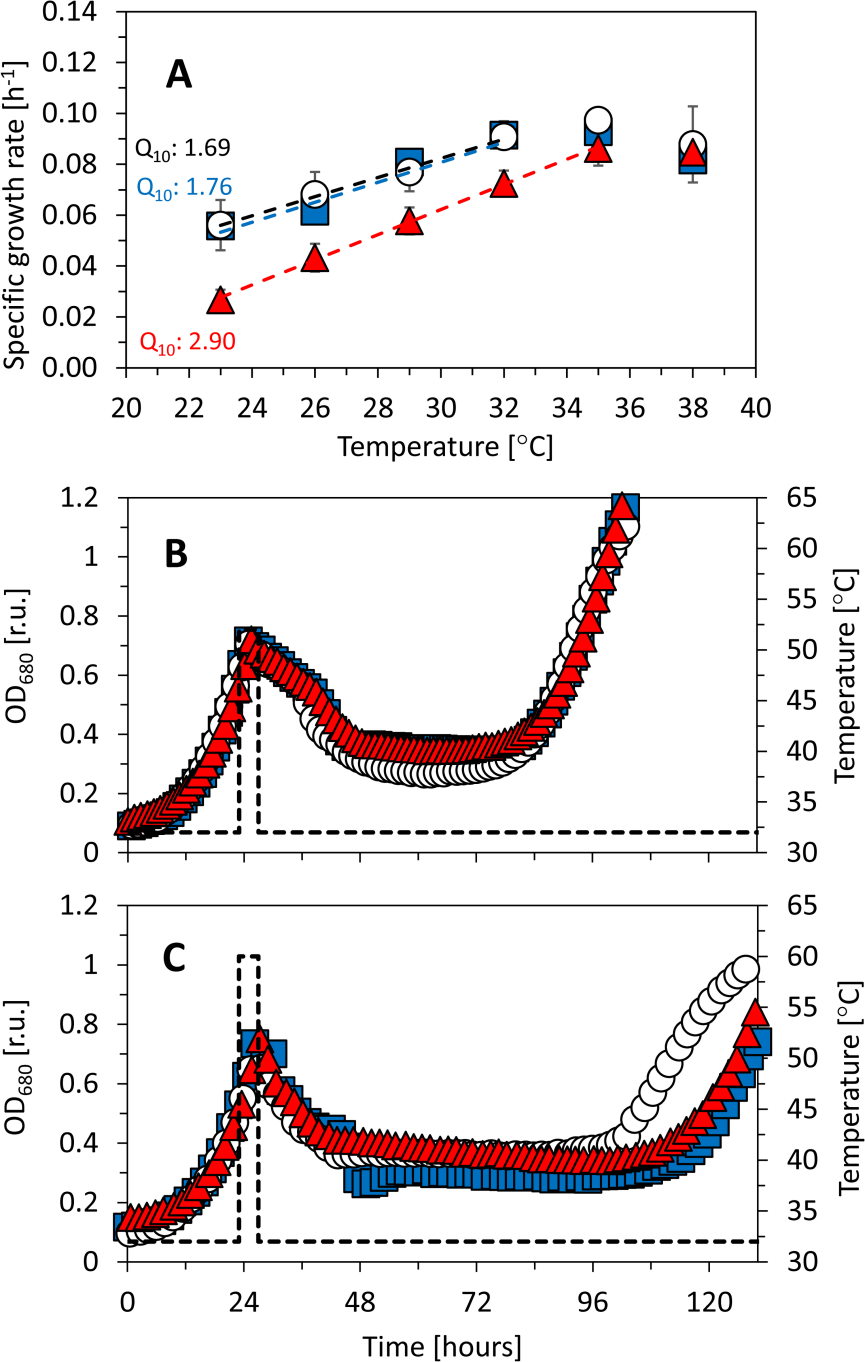
Growth rates of *Synechocystis* sp. PCC 6803 substrains GT-L (white circles), GT-B (red triangles) and PCC-B (blue squares) under temperatures 23°C—38°C during cultivation in a quasi-continuous regime (A) and after introduction of heat shock of 52°C (B) and 60°C (C) during the batch growth. A–The Q_10_ coefficient was calculated according to [19], the dashed lines represent linear fitting of the data points by the least squares method in the temperature range 23°C—32°C (23°C—35°C in case of the substrain GT-B). Each point represents average of at least four independent experiments, error bars represent standard errors. B, C—Batch growth was performed at 32°C and the heat shock was introduced by increasing temperature for four hours after the cultures reached OD_680_ 0.7 (the temperature record is marked by dashed line). The batch experiments were performed at least in three biological replicates with quantitatively similar results, data from one representative experiment are shown.

Growth of the three *Synechocystis* substrains was also tested at temperatures below and above the growth optimum. During the low temperature experiments, *Synechocystis* cultures were cultivated in the quasi-continuous regime in a temperature range 23–35°C (***Fig 5A***) under 220 *μ*mol(photons) m^-2^ s^-1^ of red light (with addition of 25 *μ*mol(photons) m^-2^ s^-1^ of blue light) as a light intensity that saturated growth of all *Synechocystis* substrains from more than 95% at 32°C (***Fig 2A***). Optimal growth temperature of 35°C was identical for all tested *Synechocystis* substrains. However, *Synechocystis* GT-B was more sensitive to temperatures below the growth optimum (23–32°C) than the other two substrains. At 32°C, where growth of both substrains GT-L and PCC-B was already saturated from more than 93%, the substrain GT-B reached only 84% of growth rates maximum. This is consistent with the observation that under 220 *μ*mol(photons) m^-2^ s^-1^ of red light (with addition of 25 *μ*mol(photons) m^-2^ s^-1^ of blue light) and at 32°C, growth rates of substrain GT-B were reduced when compared to the other two substrains (***Fig 2A***). Sequential stepwise temperature reduction from 32°C to 23°C resulted in very similar response of substrains GT-L and PCC-B: growth rates of both these substrains were decreasing with similar temperature coefficients Q_10_ 1.69 and 1.76, respectively. However, the temperature coefficient Q_10_ of substrain GT-B was measured as 2.90 (***Fig 5A***), which indicates increased sensitivity to low temperature in this substrain.

#### High temperature

Additionally, the effect of temperature higher than the growth optimum on *Synechocystis* growth was tested. At first, all three *Synechocystis* substrains were cultivated in the quasi-continuous regime at 38°C, where only minimal variability in growth rates was observed (***Fig 5A***). At higher temperatures it was not possible to cultivate *Synechocystis* in the quasi-continuous regime anymore. Therefore, at higher temperatures, the cultures were cultivated in a batch regime at 32°C and only short term (four hours) heat shock was introduced by increasing temperature from 32°C to 52°C or to 60°C, respectively, once the cultures reached optical density OD_680_ 0.7 (approximately 4 x 10^7^ cells ml^-1^).

All three *Synechocystis* substrains were able to recover from both four hours heat shocks at 52°C and 60°C. After the 52°C treatment, all tested substrains recovered growth after c. 72 hours (***Fig 5B***). After the 60°C treatment, the substrain GT-L recovered growth after c. 86 hours, whereas the other two substrains recovered growth after c. 100 hours (***Fig 5C***).

### *Synechocystis* sensitivity to salt stress

**Fig 6:**
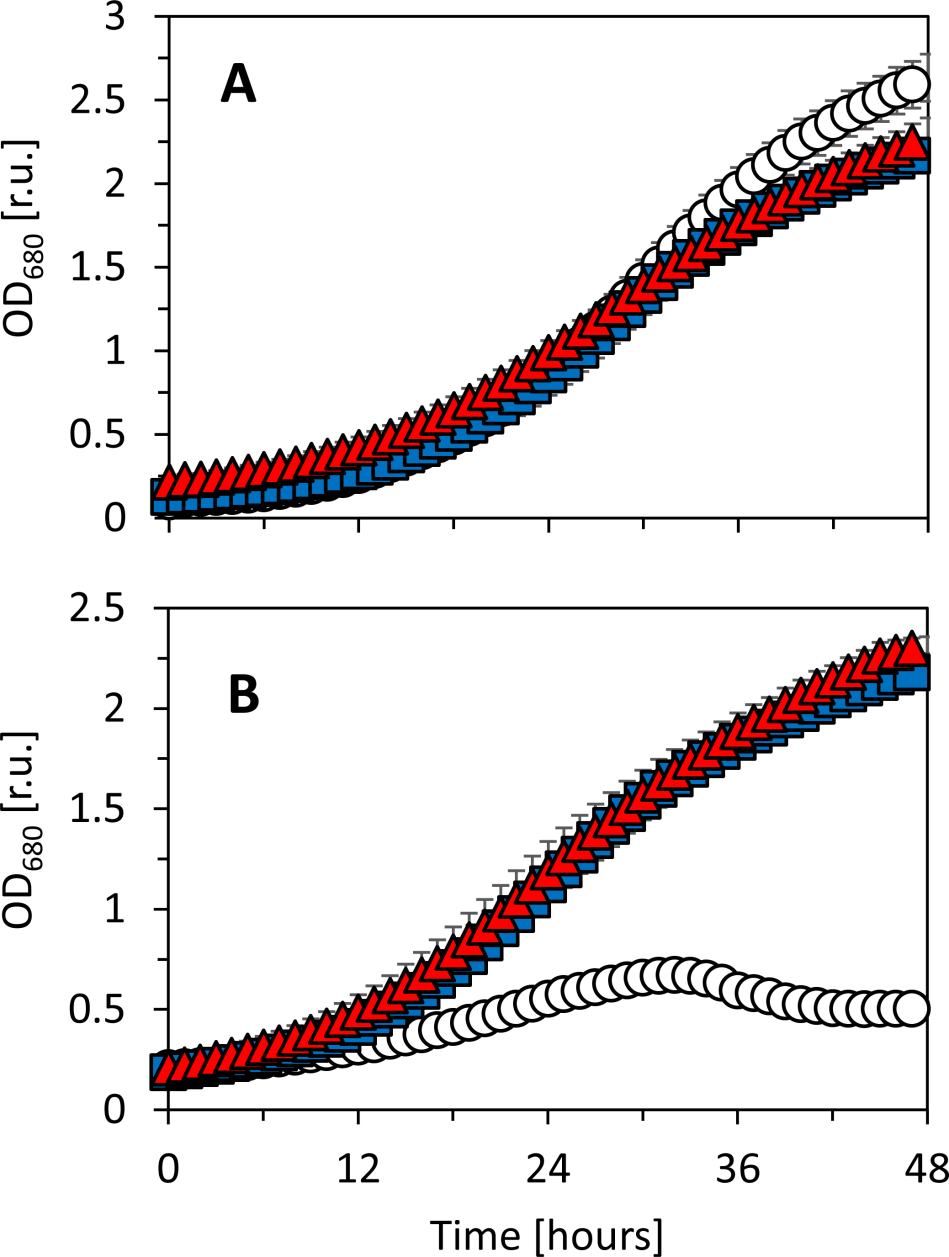
Batch growth of *Synechocystis* sp. PCC 6803 substrains GT-L (white circles), GT-B (red triangles) and PCC-B (blue squares) in BG11 medium (A) and BG11 medium supplemented with 0.5 M NaCl at the beginning of the batch cultivation (B). The plotted values represent averages of at least three independent experiments, the error bars represent standard errors.

*Synechocystis* was also cultivated in a batch regime in the presence of 0.5 M NaCl in the cultivation medium BG11. *Synechocystis* substrains GT-B and PCC-B showed only slight differences in growth in standard BG11 medium and in BG11 enriched with NaCl (***Fig 6***). On the contrary, substrain GT-L was not able to grow in the presence of NaCl. Concerning overall robustness of GT-L substrain, this was quite surprising since this substrain was not sensitive to any previously tested condition, including high intensities of red, blue and white light and low and high temperatures. On the contrary, under high light, this substrain showed significantly better fitness than the other two tested substrains.

The sensitivity of substrain GT-L to the presence of NaCl in the cultivation media was further investigated. Salt stress in cyanobacteria affects, among other processes, homeostasis of inorganic ions [24]. Therefore, in order to test growth restoration in the presence of NaCl, growth of *Synechocystis* GT-L was tested in the batch regime in BG11 media enriched with MgSO_4_, K_2_HPO_4_, and CaCl_2_ (or with combinations of these nutrients). Full grow curve of *Synechocystis* GT-L in the presence of NaCl was restored after addition of CaCl_2_ together with K_2_HPO_4_ in the concentration five times higher than in standard BG11 medium (Fig L in S1 File). However, growth in the quasi-continuous regime always ceased soon after the first dilution by the cultivation media enriched by salts. Therefore, to reach stable growth in the quasi-continuous regime, it was necessary to cultivate *Synechocystis* GT-L in full 5xBG11 medium that contained each single nutrient at concentrations five times higher than regular BG11. Even in 5xBG11, growth rates of *Synechocystis* GT-L in the presence of NaCl reached only 60% of the growth rates as in standard BG11 medium (0.048±0.002 h^-1^ and 0.079±0.011 h^-1^, respectively, Fig M in S1 File).

## Discussion

All *Synechocystis* substrains were not sensitive to the presence of glucose in both liquid and solid cultivation media during mixotrophic cultivation on light. Both GT-L and GT-B substrains were expected to be glucose-tolerant since they represent relatives of the first derived glucose-tolerant *Synechocystis* substrain “GT” (Williams (1988) [16], ***Fig 1***). However, *Synechocystis* 6803 in Pasteur Culture Collection was reported previously to be glucose sensitive [7], even though glucose tolerance was observed in another substrain derived from Pasteur Culture Collection, PCC-M [10]. The newly discovered glucose tolerance of the PCC-B substrain reported in this study can be related to a spontaneous glucose tolerance switch as already described previously [7]. The ability of mixotrophic growth in *Synechocystis* is a complex process that can be defined by several genes (such as *pmgA* [25], *infA* or *glcP* [10]), transcription factors (such as Sll0822 [10]) but also by post-translational modifications or modulation of enzyme activities [26]. To reveal nature of glucose tolerance in the substrain PCC-B, further investigation is needed that would take into consideration these factors.

Under modest cultivation conditions (low light, temperature 32°C and increased carbon concentration), only small differences in almost all measured parameters were detected among the tested *Synechocystis* substrains, including growth rates (***Fig 2***), photosynthesis (***Fig 3***, Fig E in S1 File), content of cellular saccharides, cell size and cellular dry weight (***Fig 4***, Fig I and J in S1 File), CO_2_ fixation (Fig D in S1 File), and pigments content (Fig F in S1 File). This is mostly consistent with results of the previous study where only minor differences in growth rates, oxygen evolution and total chlorophyll levels were detected among *Synechocystis* substrains GT-Kazusa, GT-O1 and PCC-Moscow under “standard” laboratory conditions (low light, 30°C, ambient CO_2_) [15]. On the other hand, even under modest conditions as used in this study, content of cellular proteins was elevated in substrain PCC-B (Fig G in S1 File). Similarly, variations in cell size, whole-cell absorption and 77 K fluorescence was reported previously under “standard” conditions [15]. This suggests that some physiology variation among *Synechocystis* substrains can be expected even under “standard” conditions, however, the phenotypic differences as detected in this study were much more pronounced under intensive and stress cultivation conditions (such as high light, low temperature or in the presence of salt in the cultivation medium).

In general, the most sensitive *Synechocystis* substrain identified in this study was the substrain GT-B. Growth rates of this substrain were inhibited under high intensities of red light (585–670 nm) and even under low intensities of non-red and non-blue part of white light spectra (485–585 nm and 670–700 nm) after addition of these photons to growth saturating red light (similarly as PCC-B substrain, ***Fig 2***). Growth reduction of GT-B during sequential stepwise temperature reduction from 32°C to 23°C was also pronounced more than in the other two tested substrains (***Fig 5***). Under high red light, *Synechocystis* GT-B reduced photosynthetic capacity (***Fig 3***), and the changes in cell size, dry weight and content of both total and storage carbohydrates were much less pronounced when compared to the other two tested substrains (***Fig 4***, Figs I and J in S1 File). In addition to other changes, the S-M fluorescence rise was reduced in this substrain under high red light (***Fig 3***). The S-M rise was shown previously to be connected with state 2 to state 1 transition and it was also suggested that it reflects a protective mechanism for excess energy dissipation [27]. Based on reduction of the fluorescence rise during the S-M transition in substrain GT-B, it can be expected that the decreased efficiency of red photons utilization (as reflected by the previously mentioned GT-B characteristics) was connected with reduced capacity of energy transfer from phycobilisomes antenna to photosystem II and/or with reduced photoprotection capacities in this substrain.

On the contrary, *Synechocystis* GT-L and PCC-B used red photons with better efficiency when compared to GT-B. Under high red light, growth of *Synechocystis* GT-L and PCC-B was not inhibited and these substrains also accumulated higher amount of carbohydrates in the cells (***Fig 4*** and Fig I in S1 File). Content of glycogen in *Synechocystis* was determined previously up to 11.1±0.3% (of cellular dry weight) after 15 days of cultivation in another glucose tolerant substrain [28], or up to 67.5±3.7 *μ*g(glycogen) *μ*g(chl)^-1^ after 48 hours under nitrogen starvation [29]. In this study, substrain GT-L was able to accumulate up to 15.8±2.5% or 15.9±1.8 *μ*g(glycogen) *μ*g(chl)^-1^ of glycogen during exponential growth. The other two tested substrains accumulated always below 10% of glycogen in the cells. Even though the substrain GT-L looks as promising candidate substrain for glycogen production, comparison with the previous studies is difficult since the content of glycogen was strongly dependent on light availability (***Fig 4*** and Fig J in S1 File) that was lower in the previous studies [28,30].

The tested *Synechocystis* substrains varied also in growth rates (***Fig 2***), photosynthetic rates (***Fig 3***), cellular dry weight (***Fig 4***), proteins content (Fig G in S1 File) and carbohydrates content (***Fig 4***, Figs I and J in S1 File). Such variation on *Synechocystis* substrain level is reported for the first time here. The overall carbohydrates content (up to c. 40% of dry weight for GT-L substrain under high red light) was higher than reported previously in another *Synechocystis* substrain (up to c. 13%, [31]). On the other hand, cellular protein content (32–57% of cellular dry weight for all three *Synechocystis* substrains tested here) was lower than reported previously (61–73%, [31]), even though the photosynthetic rates measured here were comparable with the previous study (0.03–0.21 mol(O_2_) mol(Chl^-1^) s^-1^ in this study vs. 0.066–0.081 mol(O_2_) mol(Chl^-1^) s^-1^ in Touloupakis et al. (2015) [31]). We also observed differences in cell size. Substrain GT-L that was having cells of the biggest size under high red light (the difference in cell size between GT-L and GT-B under the highest tested red light was 48%). Differences in cell size were observed previously among *Synechocystis* substrains GT-O1 and GT-Kazusa and PCC-Moscow [15]. The cell size variation as observed here is consistent with variations in the content of cellular saccharides and cellular dry weight (***Fig 4***, Figs I and J in S1 File).

The observed phenotypic variation shows that the effectivity of cellular energy management can vary among *Synechocystis* substrains, which can lead to substantial changes in cellular composition. Therefore, considering *Synechocystis* sp. PCC 6803 as a candidate strain for optimization of synthesis of targeted compounds (such as glycogen or proteins, but probably also for other products) can be tricky since 1) some *Synechocystis* substrains are more sensitive to specific cultivation conditions than others, 2) some *Synechocystis* substrains can produce specific compounds with higher rates than others, and 3) it cannot be excluded that *Synechocystis* substrains can “spontaneously” change phenotype in time.

Growth rates of all *Synechocystis* substrains increased only slightly after addition of blue photons along to the saturating red light (***Fig 2***). This fact can be connected with activation of non-photochemical quenching in *Synechocystis* under blue light [32]. On the other hand, addition of non-blue and non-red photons (485–585 nm and 670–700 nm) to saturating red light inhibited growth of substrains GT-B and PCC-B but increased growth rates of substrain GT-L (***Fig 2***). Interestingly, the fluorescence induction had similar trends as under red light only (***Fig 3***), which suggests distinct mechanisms of growth inhibition under red photons and photons of wavelengths 485–585 nm and 670–700 nm. Since addition of even small amount of non-blue and non-red photons resulted in reduction of growth rates in substrain PCC-B (Fig K in S1 File), it can be expected that sensitivity to these wavelengths had a signaling character. Light quality has been previously connected with stress responses activation in *Synechocystis* [33]. Under increasing light intensities, *Synechocystis* can be expected to change intracellular redox state or increase formation of reactive oxygen species. Both these processes can then act as stress response triggers [34]. Moreover, it has been shown that genotypic differences among *Synechocystis* substrains can include modification of various histidine kinases [9–11,13]. Changes of stress signal transduction cascade together with “activated” stress response under high light can then have diverse consequences. Although further experiments are needed to map probable variations in the signaling network among *Synechocystis* substrains, it is demonstrated here that sensitivity to specific wavelengths can vary in *Synechocystis*. This should be taken into consideration in studies that address effect of light on *Synechocystis* physiology or in studies and applications that aim on phototrophic production optimization.

Growth rates of substrain GT-B between 23°C–32°C were more reduced in comparison to the two other two substrains (***Fig 5***). *Synechocystis* sensitivity to cold stress was previously connected with saturation status of membrane lipids [35]. However, fatty acids composition was almost identical for all tested *Synechocystis* substrains at both temperatures 32°C and 23°C (Table B in S1 File). The fatty acids ratio at both 32°C and 23°C was similar as reported previously, even though some fatty acids (including 16:0, 16:1, 18:0, 18:1, 18:2, 18:3 and 18:4) were detected in slightly different concentrations when compared to the previous studies [35–37]. Change of membrane fluidity is not the only way how cyanobacteria can react to low temperatures—also formation of reactive oxygen species, changes in DNA supercoiling or cellular redox status can be detected as part of cyanobacteria cold stress response [38]. Spectrum of cold-induced genes is broad and it covers genes involved in signal perception and transduction, transcription and translation, RNA-binding proteins genes, and some genes of other functions. Moreover, several signaling proteins were described to be involved in cyanobacteria cold stress response, including universal stress histidine kinases such as Hik33. This protein can sense also red light [39] and it was even suggested that it can work as an additional red light sensor [33]. From this perspective, it can be expected that the increased sensitivity to low temperature of substrain GT-B (***Fig 5***) is connected with early red light photoinhibition of this substrain, with Hik33 being involved as a common element in both stress responses. This is consistent with our suggestion of reduced photoprotection in substrain GT-B under high red light.

Under high temperatures (52°C and 60°C) it was not possible to cultivate *Synechocystis* cultures in the quasi-continuous regime anymore and the heat stress was applied only for period of four hours during the batch cultivation (***Fig 5***). It has been previously shown that *Synechocystis* can survive heat shock of 44°C applied for 24 hours [40] or even for four days [41]. However, it was also reported that even five minutes of incubation at 52°C and 54°C is lethal for *Synechocystis* cells [42], even though in another study *Synechocystis* was found to survive for 30 minutes at 50°C [43]. Our finding of successful growth recovery of all three tested substrains from four hours heat shock at both 52°C and 60°C are not in contradiction with the previous findings. In fact, the cultures recovery after the 5 min heat shock in the previous study at both 52°C and 54°C was tested only for up to 35–40 hours [42], whereas here, the cultures recovery was followed for at least 72 hours. Successful growth recovery after 60°C heat shock as reported here shows for the first time that *Synechocystis* is able to survive such high temperature treatment.

Interestingly, the substrain GT-L (which was in general the most robust substrain under high lights and suboptimal temperatures, Figs 2–5) was not able to grow under increased salinity in standard BG11 medium (***Fig 6***). Long-term salt stress (in range of hours or days) in *Synechocystis* was reported to initiate production of compatible solutes, alteration in fatty acids composition [44] and ion transport [45], induction of specific genes [46], and accumulation of specific proteins [47]. Successful growth recovery in salt media after addition of both CaCl_2_ and K_2_HPO_4_ (Fig L in S1 File) to the substrain GT-L culture suggests that the ions management was affected under increased concentration of NaCl—both calcium and potassium ions participate in the salt stress response in cyanobacteria [24,48], and calcium was even found to be released from the cells as a consequence of membrane depolarization [49]. The finding that salt tolerance in *Synechocystis* (which was originally isolated from fresh water lake) can vary on the substrain level can have consequences for large scale production systems that consider use of sea water for cyanobacteria cultivation.

To conclude, the results presented in this study bring new insight into our understanding to *Synechocystis* sp. PCC 6803 stress sensitivity and tolerance, and undertake strongly the previous recommendation of taking into consideration specific lineages of this model strain. Based on the distribution of stress sensitivity among tested *Synechocystis* substrains, it is unlikely that stress tolerance is conserved in each specific *Synechocystis* lineage. It can be rather expected that the stress sensitivity can change spontaneously in *Synechocystis*, similarly as glucose tolerance or motility. Mechanisms of stress sensitivity “switch” on the molecular level still remain to be understood. However, the strong phenotypic variation on the substrain level represents important component of *Synechocystis* complexity that should be taken into account for keeping this model strain robust for the diverse portfolio of already started research activities, and also for its utilization in industrial applications.

## Supporting information

S1 File **Figs A–M, Tables A–B.** Supporting information that contains supporting figures, tables, methods and references.
(DOCX)

## References

[1] Sarsekeyeva F, Zayadan BK, Usserbaeva A, Bedbenov VS, Sinetova MA, Los DA. Cyanofuels: Biofuels from cyanobacteria. Reality and perspectives. Photosynth Res. Springer Netherlands; 2015;125: 329–340. doi: 10.1007/s11120-015-0103-3 25702086 10.1007/s11120-015-0103-3

[2] Wijffels RH, Kruse O, Hellingwerf KJ. Potential of industrial biotechnology with cyanobacteria and eukaryotic microalgae. Curr Opin Biotechnol. 2013;24: 405–13. doi: 10.1016/j.copbio.2013.04.004 23647970 10.1016/j.copbio.2013.04.004

[3] Kaneko T, Tanaka a, Sato S, Kotani H, Sazuka T, Miyajima N, et al Sequence analysis of the genome of the unicellular cyanobacterium Synechocystis sp. strain PCC6803. I. Sequence features in the 1 Mb region from map positions 64% to 92% of the genome. DNA Res. 1995;2: 153–166, 191–198. doi: 10.1093/dnares/2.4.153 8590279 10.1093/dnares/2.4.153

[4] Kaneko T, Sato S, Kotani H, Tanaka A, Asamizu E, Nakamura Y, et al Sequence analysis of the genome of the unicellular cyanobacterium Synechocystis sp. strain PCC6803. II. Sequence determination of the entire genome and assignment of potential protein-coding regions. DNA Res. 1996;3: 109–136. doi: 10.1093/dnares/3.3.109 8905231 10.1093/dnares/3.3.109

[5] Grigorieva G, Shestakov S. Transformation in the cyanobacterium Synechocystis sp. 6803. FEMS Microbiol Lett. 1982;13: 367–370. Available: http://onlinelibrary.wiley.com/doi/10.1111/j.1574-6968.1982.tb08289.x/full

[6] Stanier RY, Kunisawa R, Mandel M, Cohen-Bazire G. Purification and properties of unicellular blue-green algae (order Chroococcales). Bacteriol Rev. 1971;35: 171–205. Available: http://www.pubmedcentral.nih.gov/articlerender.fcgi?artid=378380&tool=pmcentrez&rendertype=abstract 4998365 10.1128/br.35.2.171-205.1971 PMC378380

[7] Ikeuchi M, Tabata S. Synechocystis sp. PCC 6803—a useful tool in the study of the genetics of cyanobacteria. Photosynth Res. 2001;70: 73–83. doi: 10.1023/A:1013887908680 16228363 10.1023/A:1013887908680

[8] Tajima N, Sato S, Maruyama F, Kaneko T, Sasaki N V, Kurokawa K, et al Genomic structure of the cyanobacterium Synechocystis sp. PCC 6803 strain GT-S. DNA Res. 2011;18: 393–9. doi: 10.1093/dnares/dsr026 21803841 10.1093/dnares/dsr026 PMC3190959

[9] Morris J, Crawford T, Jeffs a, Stockwell P, Eaton-Rye J, Summerfield T. Whole genome re-sequencing of two “wild-type” strains of the model cyanobacterium Synechocystis sp. PCC 6803. New Zeal J Bot. Taylor & Francis; 2014;52: 36–47. doi: 10.1080/0028825X.2013.846267

[10] Trautmann D, Voss B, Wilde A, Al-Babili S, Hess WR. Microevolution in cyanobacteria: re-sequencing a motile substrain of Synechocystis sp. PCC 6803. DNA Res. 2012;19: 435–48. doi: 10.1093/dnares/dss024 23069868 10.1093/dnares/dss024 PMC3514855

[11] Kanesaki Y, Shiwa Y, Tajima N, Suzuki M, Watanabe S, Sato N, et al Identification of substrain-specific mutations by massively parallel whole-genome resequencing of Synechocystis sp. PCC 6803. DNA Res. 2012;19: 67–79. doi: 10.1093/dnares/dsr042 22193367 10.1093/dnares/dsr042 PMC3276265

[12] Tichý M, Bečková M, Kopečná J, Noda J, Sobotka R, Komenda J. Strain of Synechocystis PCC 6803 with Aberrant Assembly of Photosystem II Contains Tandem Duplication of a Large Chromosomal Region. Front Plant Sci. 2016;7: 1–10. doi: 10.3389/fpls.2016.00648 27242849 10.3389/fpls.2016.00648 PMC4867675

[13] Ding Q, Chen G, Wang Y, Wei D. Identification of specific variations in a non-motile strain of cyanobacterium synechocystis sp. PCC 6803 originated from ATCC 27184 by whole genome resequencing. Int J Mol Sci. 2015;16: 24081–24093. doi: 10.3390/ijms161024081 26473841 10.3390/ijms161024081 PMC4632739

[14] Zerulla K, Ludt K, Soppa J. The ploidy level of Synechocystis sp. PCC 6803 is highly variable and is influenced by growth phase and by chemical and physical external parameters. Microbiology. 2016;162: 730–739. doi: 10.1099/mic.0.000264 26919857 10.1099/mic.0.000264

[15] Morris JN, Eaton-Rye JJ, Summerfield TC. Phenotypic variation in wild-type substrains of the model cyanobacterium Synechocystis sp. PCC 6803. New Zeal J Bot. 2016; doi: 10.1080/0028825X.2016.1231124

[16] Williams JGK. Construction of specific mutations in photosystem II photosynthetic reaction center by genetic engineering methods in Synechocystis 6803. Methods Enzymol. 1988;167: 766–778. doi: 10.1016/0076-6879(88)67088-1

[17] Nedbal L, Trtílek M, Cervený J, Komárek O, Pakrasi HB. A photobioreactor system for precision cultivation of photoautotrophic microorganisms and for high-content analysis of suspension dynamics. Biotechnol Bioeng. 2008;100: 902–10. doi: 10.1002/bit.21833 18383143 10.1002/bit.21833

[18] ervený J, Šetlík I, Trtílek M, Nedbal L. Photobioreactor for cultivation and real-time, in-situ measurement of O2 and CO2 exchange rates, growth dynamics, and of chlorophyll fluorescence emission of photoautotrophic microorganisms. Eng Life Sci. 2009;9: 247–253. doi: 10.1002/elsc.200800123

[19] Zavřel T, Sinetova MA, Búzová D, Literáková P, Červený J. Characterization of a model cyanobacterium Synechocystis sp. PCC 6803 autotrophic growth in a flat-panel photobioreactor. Eng Life Sci. 2015;15: 122–132. doi: 10.1002/elsc.201300165

[20] Sinetova MA, Červený J, Zavřel T, Nedbal L. On the dynamics and constraints of batch culture growth of the cyanobacterium *Cyanothece* sp. ATCC 51142. J Biotechnol. 2012;162: 148–55. doi: 10.1016/j.jbiotec.2012.04.009 22575787 10.1016/j.jbiotec.2012.04.009

[21] Zavřel T, Sinetova MA, Červený J. Measurement of Chlorophyll *a* and Carotenoids Concentration in Cyanobacteria. bio-protocol. 2015;5: 1–5. Available: http://www.bio-protocol.org/e146727617271

[22] Bennett a, Bogorad L. Complementary chromatic adaption in a filamentous blue-green alga. JCellBiol. 1973;58: 419–435. 10.1083/jcb.58.2.419 PMC2109051 4199659

[23] Dubois M, Gilles K a, Ton JKH, Rebers P a, Smith F. Colorimetric Method for Determination of Sugars and Related Substances. Anal Chem. 1956;28: 350–356. doi: 10.1021/ac60111a017

[24] Hagemann M. Molecular biology of cyanobacterial salt acclimation. FEMS Microbiol Rev. 2011;35: 87–123. doi: 10.1111/j.1574-6976.2010.00234.x 20618868 10.1111/j.1574-6976.2010.00234.x

[25] Hihara Y, Ikeuchi M. Mutation in a novel gene required for photomixotrophic growth leads to enhanced photoautotrophic growth of Synechocystis sp. PCC 6803. Photosynth Res. 1997;53: 243–252. Available: http://dx.doi.org/10.1023/A:1005879905365

[26] Kahlon S, Beeri K, Ohkawa H, Hihara Y, Murik O, Suzuki I, et al A putative sensor kinase, Hik31, is involved in the response of Synechocystis sp. strain PCC 6803 to the presence of glucose. Microbiology. 2006;152: 647–655. doi: 10.1099/mic.0.28510-0 16514145 10.1099/mic.0.28510-0

[27] Kaňa R, Kotabová E, Komárek O, Šedivá B, Papageorgiou GC, Govindjee, et al The slow S to M fluorescence rise in cyanobacteria is due to a state 2 to state 1 transition. Biochim Biophys Acta—Bioenerg. 2012;1817: 1237–1247. doi: 10.1016/j.bbabio.2012.02.024 22402228 10.1016/j.bbabio.2012.02.024

[28] Joseph A, Aikawa S, Sasaki K, Matsuda F, Hasunuma T, Kondo A. Increased biomass production and glycogen accumulation in apcE gene deleted Synechocystis sp. PCC 6803. AMB Express. 2014;4: 17 doi: 10.1186/s13568-014-0017-z 24949254 10.1186/s13568-014-0017-z PMC4052703

[29] Gründel M, Scheunemann R, Lockau W, Zilliges Y. Impaired glycogen synthesis causes metabolic overflow reactions and affects stress responses in the cyanobacterium Synechocystis sp. PCC 6803. Microbiology. 2012;158: 3032–43. doi: 10.1099/mic.0.062950-0 23038809 10.1099/mic.0.062950-0

[30] Gründel M, Scheunemann R, Lockau W, Zilliges Y. Impaired glycogen synthesis causes metabolic overflow reactions and affects stress responses in the cyanobacterium Synechocystis sp. PCC 6803. Microbiol (United Kingdom). 2012;158: 3032–3043. doi: 10.1099/mic.0.062950–0 10.1099/mic.0.062950-0 23038809

[31] Touloupakis E, Cicchi B, Torzillo G. A bioenergetic assessment of photosynthetic growth of Synechocystis sp. PCC 6803 in continuous cultures. Biotechnol Biofuels. BioMed Central; 2015;8: 133 doi: 10.1186/s13068-015-0319-7 26379769 10.1186/s13068-015-0319-7 PMC4571542

[32] Kirilovsky D, Kaňa R, Prášil O. Mechanisms Modulating Energy Arriving at Reaction Centers in Cyanobacteria. Non-Photochemical Quenching and Energy Dissipation in Plants, Algae and Cyanobacteria. 2014 doi: 10.1007/978-94-017-9032-1

[33] Mironov KS, Los DA. Light Regulation of Cold Stress Responses in Synechocystis Stress and Environmental Regulation of Gene Expression and Adaptation in Bacteria. Hoboken, NJ, USA: John Wiley & Sons, Inc.; 2016 pp. 881–889. doi: 10.1002/9781119004813.ch86

[34] Sinetova MA, Los DA. Lessons from cyanobacterial transcriptomics: Universal genes and triggers of stress responses. Mol Biol. 2016;50: 606–614. doi: 10.1134/S0026893316040117 10.7868/S002689841604011X 27668606

[35] Wada H, Murata N. Temperature-induced Changes in the Fatty Acid Composition of the Cyanobacterium, Synechocystis PCC 6803. Plant Physiol. 1990; 1062–1069. 10.1104/pp.92.4.1062 PMC1062416 16667371

[36] Dilley R a, Nishiyama Y, Gombos Z, Murata N. Bioenergetic responses of Synechocystis 6803 fatty acid desaturase mutants at low temperatures. J Bioenerg Biomembr. 2001;33: 135–41. Available: http://www.ncbi.nlm.nih.gov/pubmed/11456219 11456219 10.1023/a:1010752531909

[37] Nanjo Y, Mizusawa N, Wada H, Slabas AR, Hayashi H, Nishiyama Y. Synthesis of fatty acids de novo is required for photosynthetic acclimation of Synechocystis sp. PCC 6803 to high temperature. Biochim Biophys Acta. Elsevier B.V.; 2010;1797: 1483–90. doi: 10.1016/j.bbabio.2010.03.014 20303926 10.1016/j.bbabio.2010.03.014

[38] Sinetova MA, Los DA. New insights in cyanobacterial cold stress responses: Genes, sensors, and molecular triggers. Biochim Biophys Acta—Gen Subj. Elsevier B.V.; 2016;1860: 2391–2403. doi: 10.1016/j.bbagen.2016.07.006 27422804 10.1016/j.bbagen.2016.07.006

[39] Kreslavski VD, Fomina IR, Los DA, Carpentier R, Kuznetsov V V., Allakhverdiev SI. Red and near infra-red signaling: Hypothesis and perspectives. J Photochem Photobiol C Photochem Rev. 2012;13: 190–203. doi: 10.1016/j.jphotochemrev.2012.01.002

[40] Červený J, Sinetova MA, Zavřel T, Los DA. Mechanisms of high temperature resistance of Synechocystis sp. PCC 6803: An Impact of histidine kinase 34. Life. 2015;5: 676–699. doi: 10.3390/life5010676 25738257 10.3390/life5010676 PMC4390874

[41] Sheng J, Kim HW, Badalamenti JP, Zhou C, Sridharakrishnan S, Krajmalnik-Brown R, et al Effects of temperature shifts on growth rate and lipid characteristics of Synechocystis sp. PCC6803 in a bench-top photobioreactor. Bioresour Technol. Elsevier Ltd; 2011;102: 11218–25. doi: 10.1016/j.biortech.2011.09.083 22001056 10.1016/j.biortech.2011.09.083

[42] Inoue N, Taira Y, Emi T, Yamane Y, Kashino Y, Koike H, et al Acclimation to the growth temperature and the high-temperature effects on photosystem II and plasma membranes in a mesophilic cyanobacterium, Synechocystis sp. PCC6803. Plant Cell Physiol. 2001;42: 1140–8. Available: http://www.ncbi.nlm.nih.gov/pubmed/11673630 11673630 10.1093/pcp/pce147

[43] Fang F, Barnum SR. The heat shock gene, htpG, and thermotolerance in the cyanobacterium, Synechocystis sp. PCC 6803. Curr Microbiol. 2003;47: 341–346. doi: 10.1007/s00284-002-4015-z 14629017 10.1007/s00284-002-4015-z

[44] Huflejt ME, Tremolieres A, Pineau B, Lang JK, Hatheway J, Packer L. Changes in membrane lipid composition during saline growth of the fresh water cyanobacterium *Synechococcus* 6311. Plant Physiol. 1990;94: 1512–1521. doi: 10.1104/pp.94.4.1512 11537468 10.1104/pp.94.4.1512 PMC1077414

[45] Pade N, Hagemann M. Salt Acclimation of Cyanobacteria and Their Application in Biotechnology. Life. 2014;5: 25–49. doi: 10.3390/life5010025 25551682 10.3390/life5010025 PMC4390839

[46] Marin K, Kanesaki Y, Los D a, Murata N, Suzuki I, Hagemann M. Gene expression profiling reflects physiological processes in salt acclimation of Synechocystis sp. strain PCC 6803. Plant Physiol. 2004;136: 300–329. doi: 10.1104/pp.104.045047.3290 10.1104/pp.104.045047 PMC523388 15361582

[47] Fulda S, Mikkat S, Huang F, Huckauf J, Marin K, Norling B, et al Proteome analysis of salt stress response in the cyanobacterium Synechocystis sp. strain PCC 6803. Proteomics. 2006;6: 2733–2745. doi: 10.1002/pmic.200500538 16572470 10.1002/pmic.200500538

[48] Torrecilla I, Leganes F, Bonilla I, Fernandez-pinas F. Calcium transits in response to salinity and osmotic stress in the nitrogen-fix cyanobacterium Anabaena sp. PCC7120, expressing cytosolic apoaequorin. Plant Cell Environ. 2001;24: 641–648.

[49] Nazarenko L V., Andreev IM, Lyukevich AA, Pisareva T V., Los DA. Calcium release from Synechocystis cells induced by depolarization of the plasma membrane: MscL as an outward Ca2+ channel. Microbiology. 2003;149: 1147–1153. doi: 10.1099/mic.0.26074-0 12724376 10.1099/mic.0.26074-0

[50] Rippka R, Deruelles J, Waterbury JB, Herdman M, Stanier RY. Generic Assignments, Strain Histories and Properties of Pure Cultures of Cyanobacteria. Microbiology. 1979;111: 1–61. doi: 10.1099/00221287-111-1-1

[51] Platt T, Gallegos CL, Harrison WG. Photoinhibition of photosynthesis in natural assemblages of marine phytoplankton. J Mar Res. 1980;38: 687–701.

