# Supplementary material for: Phenotypic characterization of Synechocystis sp. PCC 6803 substrains reveals differences in sensitivity to abiotic stress
Source: PLoS One. 2017 Dec 7;12(12):e0189130. doi: 10.1371/journal.pone.0189130 (PMC5720811; doi:10.1371/journal.pone.0189130)
Supplement: S1 File — Figs A–M, Tables A–B. Supporting information that contains supporting figures, tables, methods and references. (DOCX) [file pone.0189130.s001.docx]

**Supplementary Information**

**Phenotypic characterization of *Synechocystis* sp. PCC 6803 substrains reveals differences in sensitivity to abiotic stress**

Tomáš Zavřel^1^, Petra Očenášová^1^, Jan Červený^1^

^1^Department of Adaptive Biotechnologies, Global Change Research Institute, Academy of Science of the Czech Republic, Brno, Czech Republic

**Results**

**
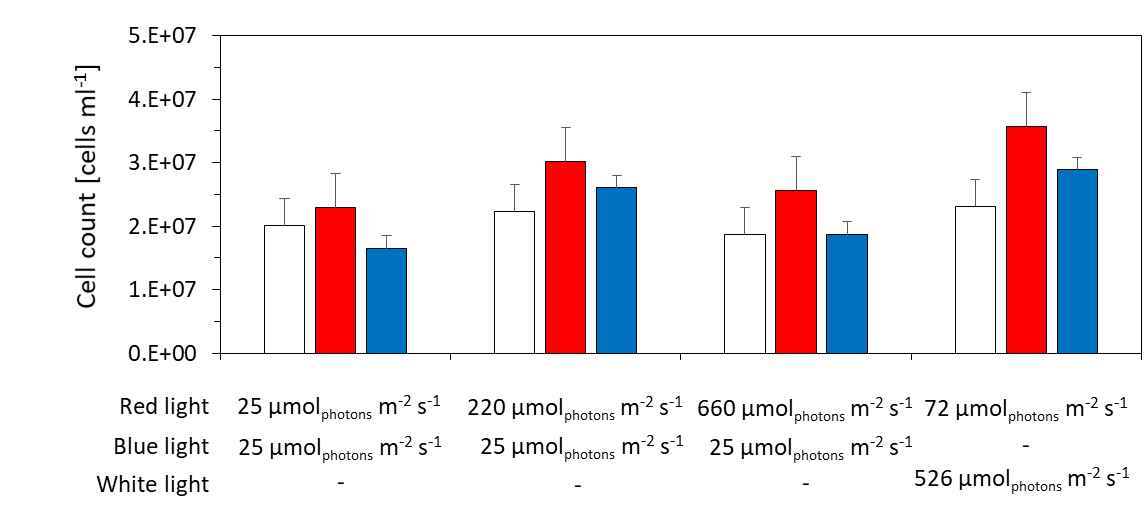
**

**Fig A** Concentration of *Synechocystis* GT-L (white bars), GT-B (red bars) and PCC-B (blue bars) cells in the culture suspensions during the quasi-continuous experiments as described in the main text. The *Synechocystis* substrains were cultivated at 32°C under input CO_2_ concentration 5 000 ppm. The values represent averages from 3–6 independent experiments, error bars represent standard errors. Variation in cell count among the tested *Synechocystis* substrains was statistically not significant (Tukey’s HSD post-hoc test following one-way ANOVA: p>0.05).


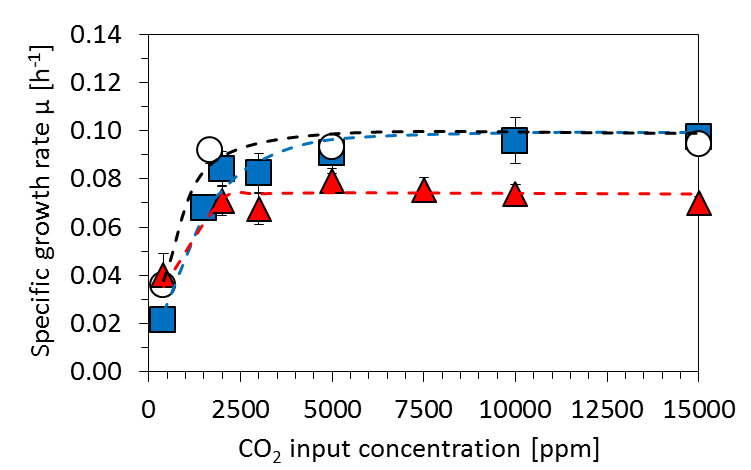


**Fig** **B** Growth rates of *Synechocystis* sp. PCC 6803 substrains GT-L (white circles), GT-BGT-B (red triangles) and PCC-B (blue squares) under input CO_2_ concentrations ranging between 400 ppm – 15 000 ppm. The cells were cultivated at 32°C under 220 µmol(photons) m^-2^ s^-1^ of red light complemented by 25 µmol(photons) m^-2^ s^-1^ of blue light in a quasi-continuous regime as described in the main text. All values represent averages of at least four independent experiments, error intervals represent standard errors. The dashed lines represent data points fits by the function derived by Platt et al. (1980) [1].

***
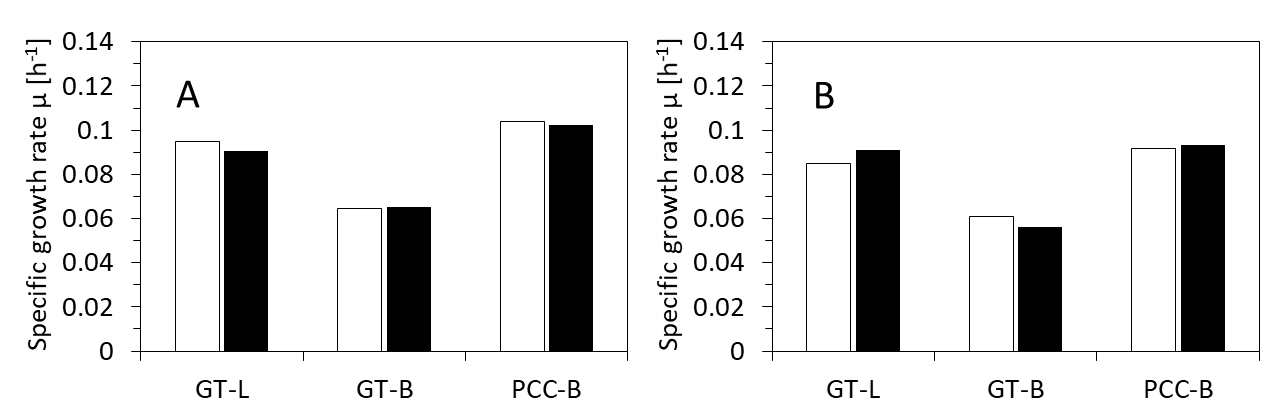
***

**Fig C** The effect of stirring (A) and bubbling (B) on specific growth rates of *Synechocystis* substrains GT-L, GT-B and PCC-B, when cultivated at 32°C under input CO_2_ concentration 5 000 ppm and under 220 µmol(photons) m^-2^ s^-1^ of red light complemented with 25 µmol(photons) m^-2^ s^-1^ of blue light (conditions saturating growth of all substrains from more than 95%), The cultures were cultivated with magnetic stirrer running at 210 rpm and with magnetic stirrer disconnected (A, white and black columns, respectively) and with bubbling rate 50 ml min^-1^ and 200 ml min^-1^ (B, white and black columns, respectively). Each experiment was performed in duplicates, average values are shown.


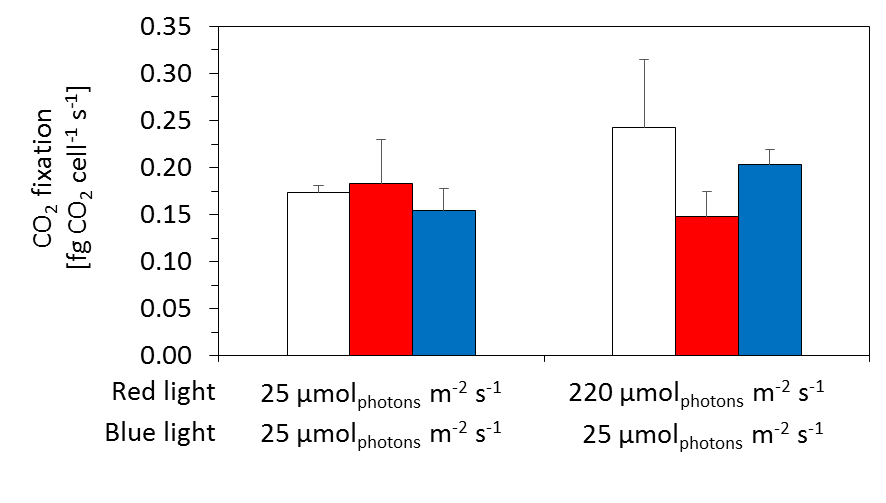


**Fig D** Carbon fixation of the *Synechocystis* sp. PCC 6803 substrains GT-L (white bars), GT-B (red bars) and PCC-B (blue bars) under 25 and 220 µmol_photons_ m^-2^ s^-1^ of red light complemented with 25 µmol(photons) m^-2^ s^-1^ of blue light. All *Synechocystis* sub-strains were cultivated at 32°C and concentration of CO_2_ in input gas 5 000 ppm in a quasi-continuous regime as described in the main text. All values represent averages of at least three independent experiments, error intervals represent standard deviations. Variation in carbon fixation among the tested *Synechocystis* substrains was statistically not significant (Tukey’s HSD post-hoc test following one-way ANOVA: p>0.05).

***
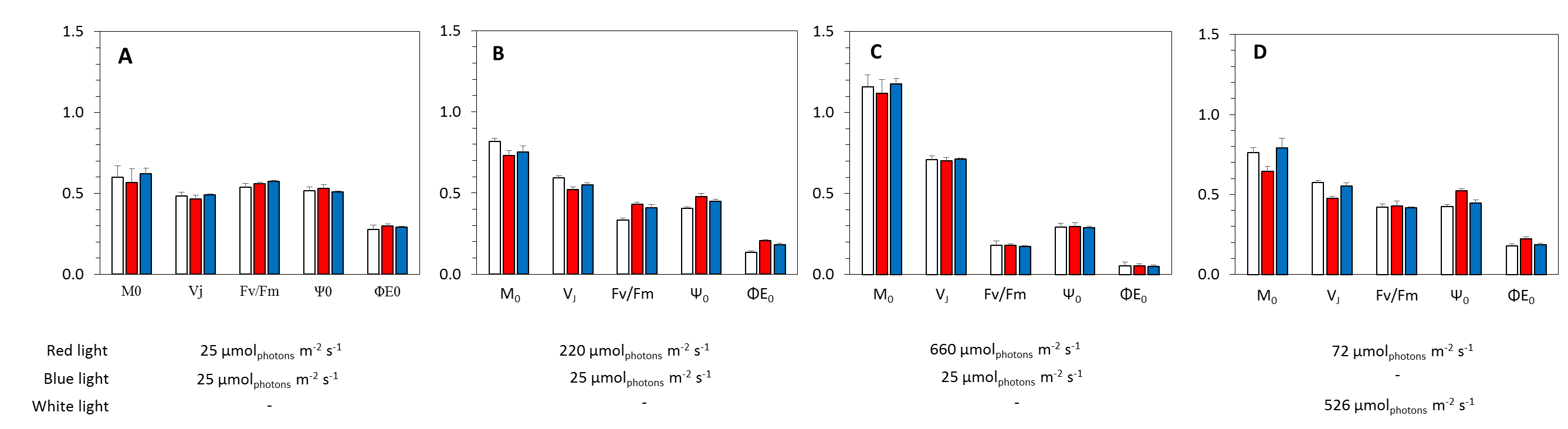
***

**Fig E** Photosynthesis induction parameters as derived from the pigment fluorescence induction kinetics (O-J-I-P) of *Synechocystis* substrains GT-L (white bars), GT-B (red bars) and PCC-B (blue bars) adapted to 25 µmol(photons) m^-2^ s^-1^ (A), 220 µmol(photons) m^-2^ s^-1^ (B) and µmol(photons) m^-2^ s^-1^ (C) of red light complemented with 25 µmol(photons) m^-2^ s^-1^ of blue light and 72 µmol(photons) m^-2^ s^-1^ of red light complemented with 526 µmol(photons) m^-2^ s^-1^ of white light (D). The calculations were based on Touloupakis et al., (2015) [2] and represent initial slope at the beginning of the variable fluorescence (M_0_), variable fluorescence at phase J (V_J_), maximal quantum yield of PSII (Fv/Fm), efficiency with which a trapped exciton can move an electron further than Q_A_^-^ into the electron transport chain (Ψ_0_) and a quantum yield of electron transport (Φ_E0_). The cells were cultivated at 32°C under input CO_2_ concentration 5 000 ppm in a quasi-continuous regime as described in the main text. All values represent averages of at least four independent experiments, error bars represent standard errors. The variation in the parameters of photosynthesis induction among the tested *Synechocystis* substrains was statistically not significant (Tukey’s HSD post-hoc test following one-way ANOVA: p>0.05).

**
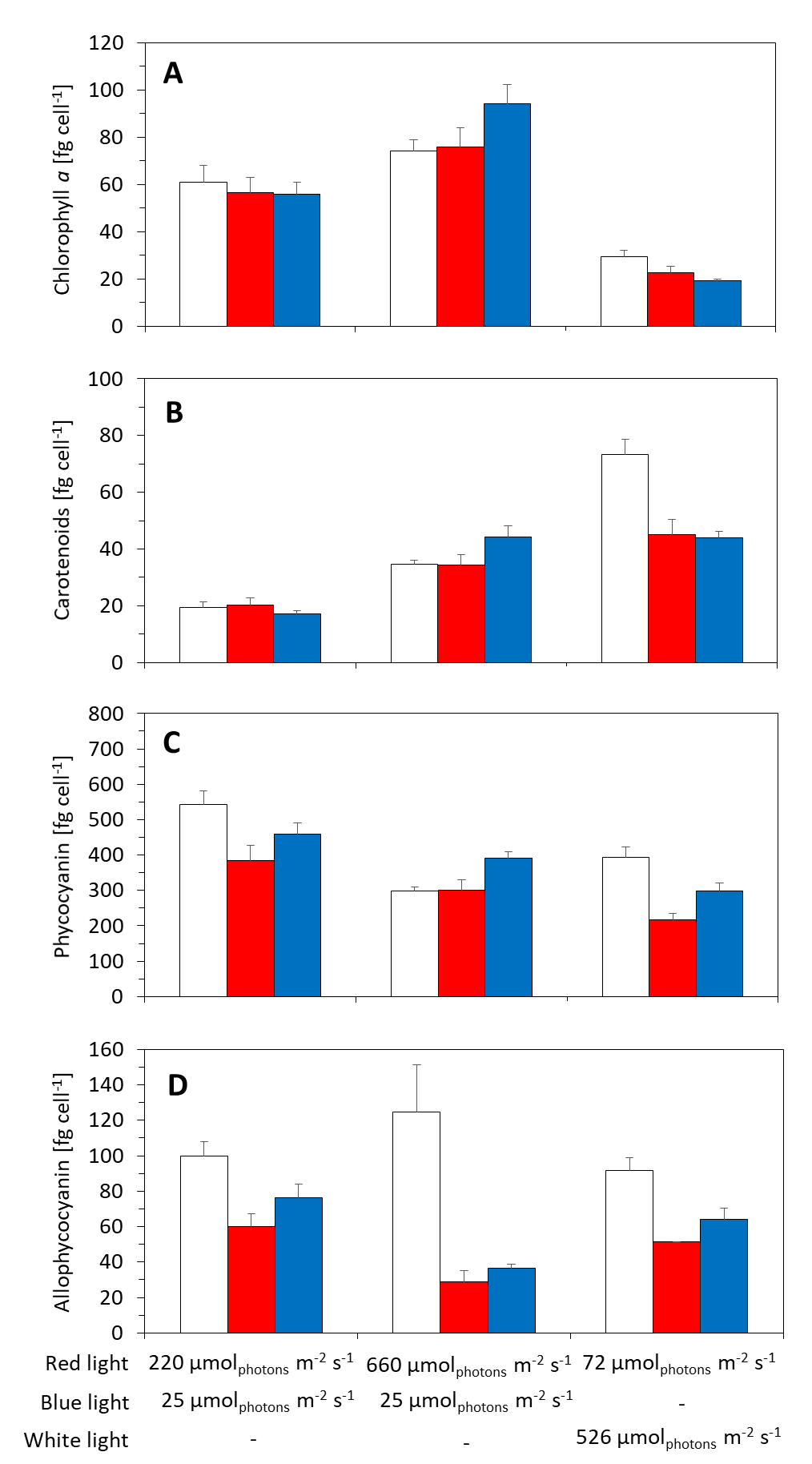
**

**Fig F** Concentrations of chlorophyll *a* (A), carotenoids (B), phycocyanin (C) and allophycocyanin (D) in *Synechocystis* substrains GT-L (white bars), GT-B (red bars) and PCC-B (blue bars) under 220 µmol(photons) m^-2^ s^-1^ and 660 µmol(photons) m^-2^ s^-1^ of red light complemented with 25 µmol(photons) m^-2^ s^-1^ of blue light and 72 µmol(photons) m^-2^ s^-1^ of red light complemented with 526 µmol(photons) m^-2^ s^-1^ of white light. All *Synechocystis* sub-strains were cultivated in a quasi-continuous regime as described in the main text at 32°C and concentration of CO_2_ in input gas 5 000 ppm. All values represent averages of at least four independent experiments, error intervals represent standard deviations. Variation in pigments concentration among the tested *Synechocystis* substrains was statistically not significant (Tukey’s HSD post-hoc test following one-way ANOVA: p>0.05).

**
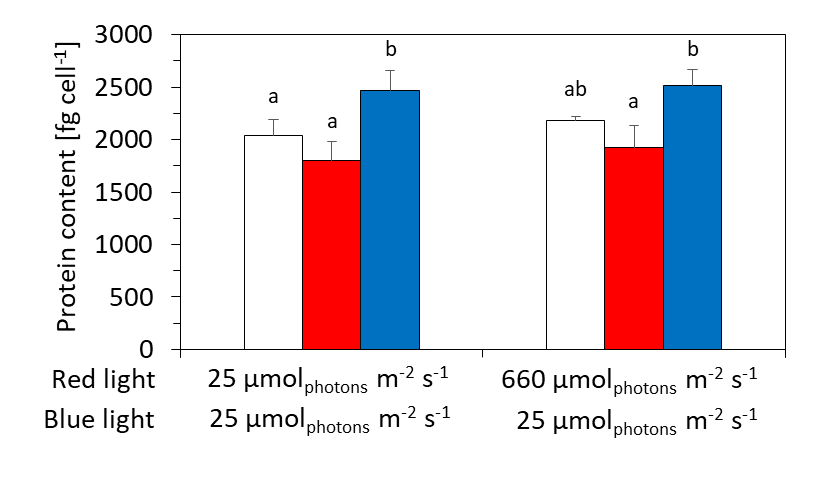
**

**Fig G** Concentrations of proteins in biomass of *Synechocystis* substrains GT-L (white bars), GT-B (red bars) and PCC-B (blue bars) adapted to 25 µmol(photons) m^-2^ s^-1^, and 660 µmol(photons) m^-2^ s^-1^ of red light complemented with 25 µmol(photons) m^-2^ s^-1^ of blue light. The cells were cultivated at 32°C under input CO_2_ concentration of 5 000 ppm in a quasi-continuous regime as described in the main text. All values represent averages of at least four independent experiments, error intervals represent standard deviations. Differences in proteins concentrations among *Synechocystis* substrains are marked by letters above the particular columns (Tukey’s’s HSD post-hoc test following one-way ANOVA: p<0.05).

**
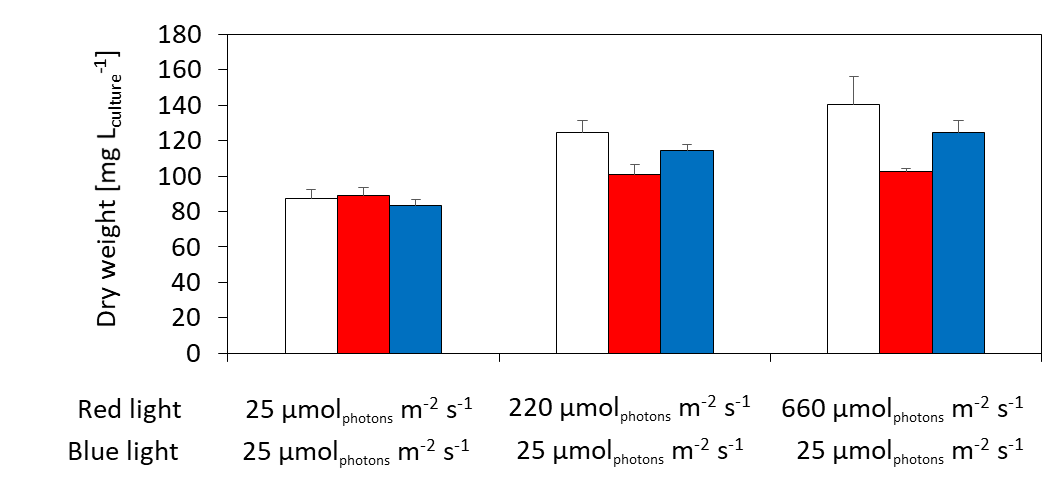
**

**Fig H** Cellular dry weight (expressed as mg L^-1^) of *Synechocystis* substrains GT-L (white bars), GT-B (red bars) and PCC-B (blue bars) adapted to 25, 220 and 660 µmol_photons_ m^-2^ s^-1^ of red light complemented with 25 µmol(photons) m^-2^ s^-1^ of blue light. The cells were cultivated at 32°C under input CO_2_ concentration of 5 000 ppm in a quasi-continuous regime as described in the main text. All values represent averages of at least four independent experiments, error bars represent standard errors. Variation in cellular dry weight (expressed as mg L^-1^) among the tested *Synechocystis* substrains was statistically not significant (Tukey’s HSD post-hoc test following one-way ANOVA: p>0.05).


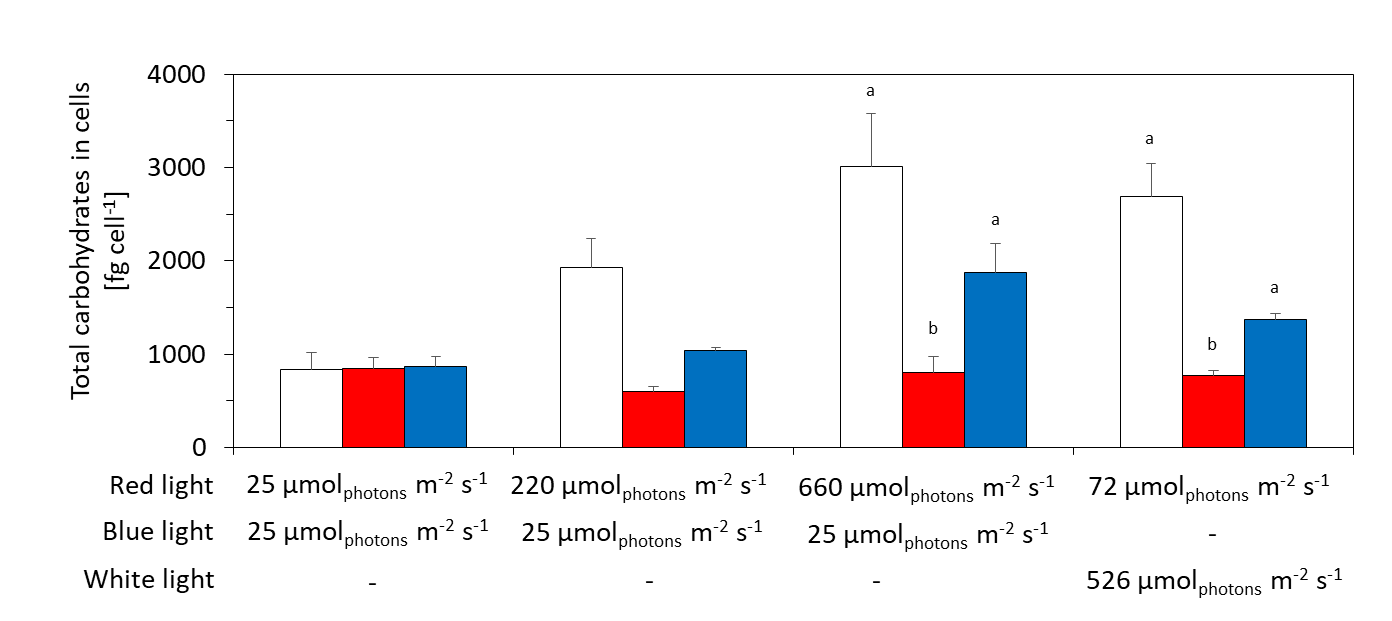


**Fig I** Concentrations of total cellular carbohydrates of *Synechocystis* substrains GT-L (white bars), GT-B (grey bars) and PCC-B (black bars) adapted to 25, 220 and 660 µmol(photons) m^-2^ s^-1^ of red light complemented with 25 µmol(photons) m^-2^ s^-1^ of blue light and 72 µmol(photons) m^-2^ s^-1^ of red light complemented with 526 µmol(photons) m^-2^ s^-1^ of white light. The cells were cultivated at 32°C under input CO_2_ concentration of 5 000 ppm in a quasi-continuous regime as described in the main text. All values represent averages of at least four independent experiments, error intervals represent standard deviations. Differences in concentrations of total saccharides among *Synechocystis* substrains are marked by letters above the particular columns (Tukey’s HSD post-hoc test following one-way ANOVA: p<0.05).

**
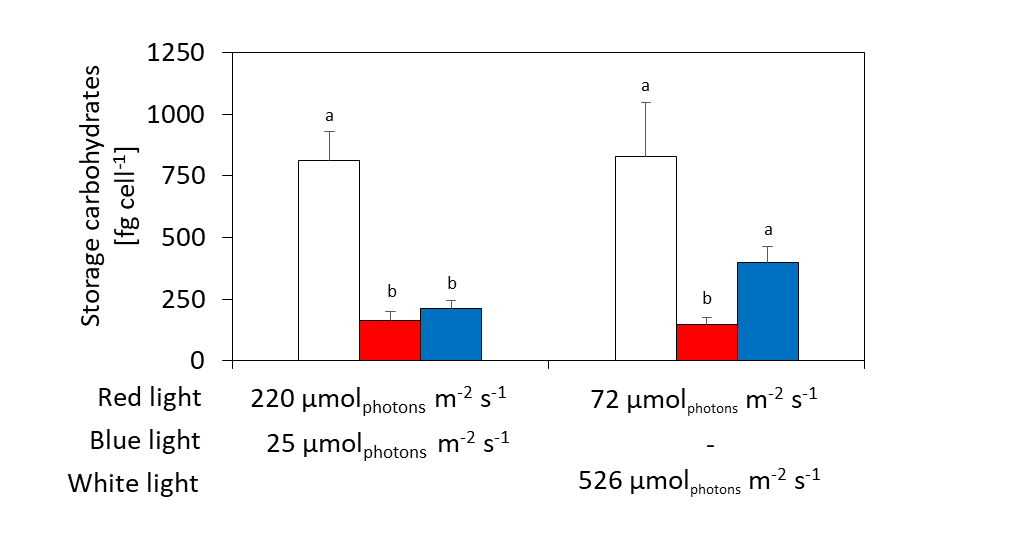
**

**Fig J** Concentrations of storage carbohydrates (glycogen) of *Synechocystis* substrains GT-L (white bars), GT-B (grey bars) and PCC-B (black bars) adapted to 220 µmol(photons) m^-2^ s^-1^ of red light complemented with 25 µmol(photons) m^-2^ s^-1^ of blue light and 72 µmol(photons) m^-2^ s^-1^ of red light complemented with 526 µmol(photons) m^-2^ s^-1^ of white light. The cells were cultivated at 32°C under input CO_2_ concentration of 5 000 ppm in a quasi-continuous regime as described in the main text. All values represent averages of at least four independent experiments, error intervals represent standard deviations. Differences in concentrations of glycogen among *Synechocystis* substrains are marked by letters above the particular columns (Tukey’s HSD post-hoc test following one-way ANOVA: p<0.05).


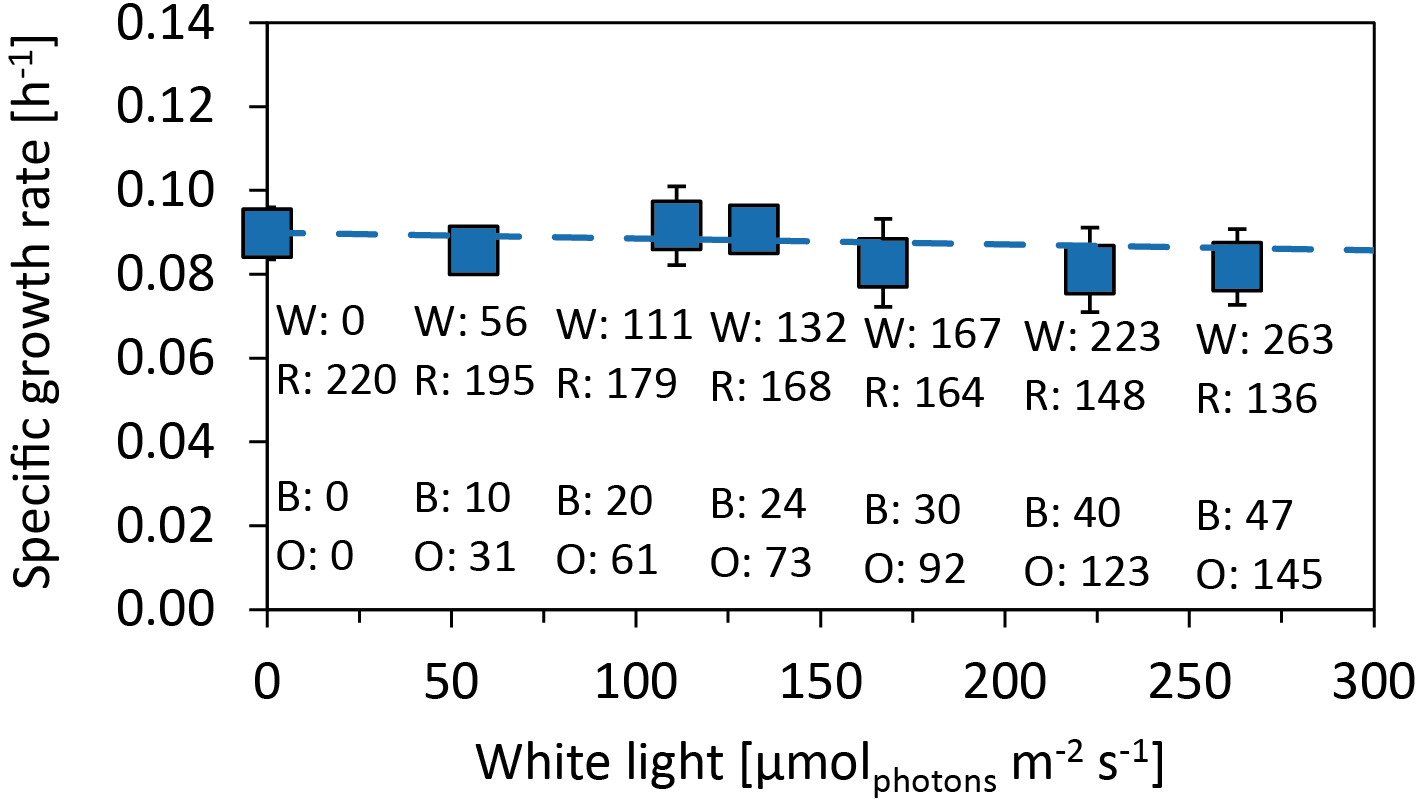


**Fig K** Growth rates of *Synechocystis* sp. PCC 6803 substrain PCC-B under white light of intensities 0 - 263 µmol(photons) m^-2^ s^-1^, complemented with red light of intensities 220-136 µmol(photons) m^-2^ s^-1^. The cells were cultivated under the same conditions as described in the caption of Figure 2 in the main text (in particular, under the same conditions as in the Figure 2D). Each point represents average of at least four independent experiments, error bars represent standard errors. W: white light intensity, R: red light intensity, B: blue photons (405 – 485 nm), O: non-red and non-blue photons (485 – 585 nm and 670 – 700 nm), all in units of µmol(photons) m^-2^ s^-1^. The dashed line represents linear fit of the data points.


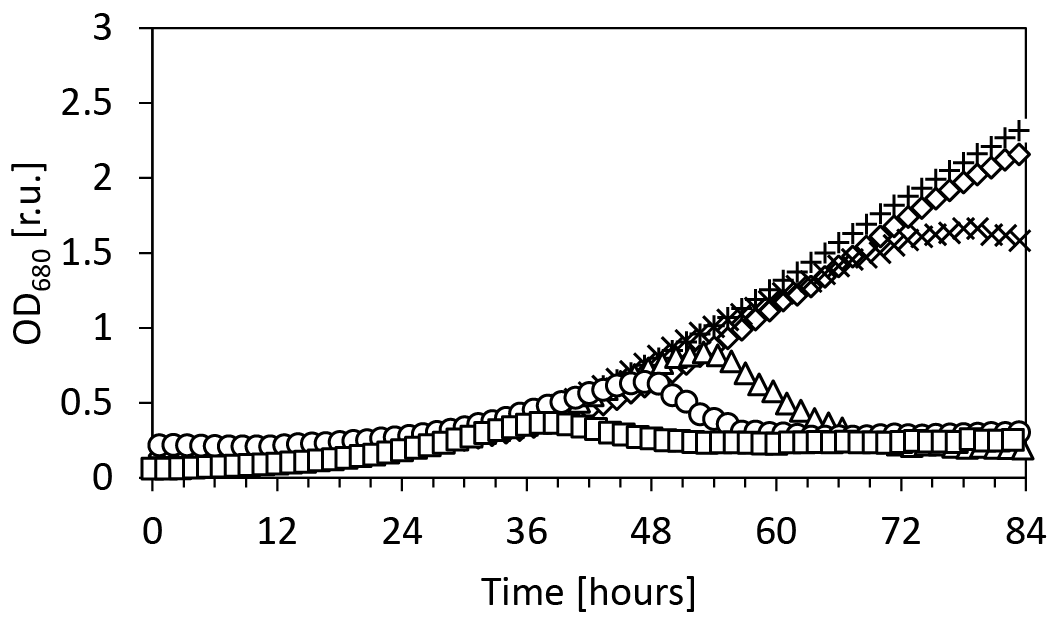


**Fig L** Batch growth of the *Synechocystis* substrain GT-L in the presence of 0.7 M NaCl in cultivation medium BG11 (○), in BG11 with 5x increased concentration of MgSO_4_ (□), 5x increased K_2_HPO_4_ (∆), 5x increased CaCl_2_ (x), 5x increased both CaCl_2_ and K_2_HPO_4_ (◊) and in BG11 containing each single nutrient in 5x higher concentration when compared to standard BG11 (+). Each experiment was performed at least in two independent replicates with qualitatively similar results, representative curves from each experiment are shown.

**
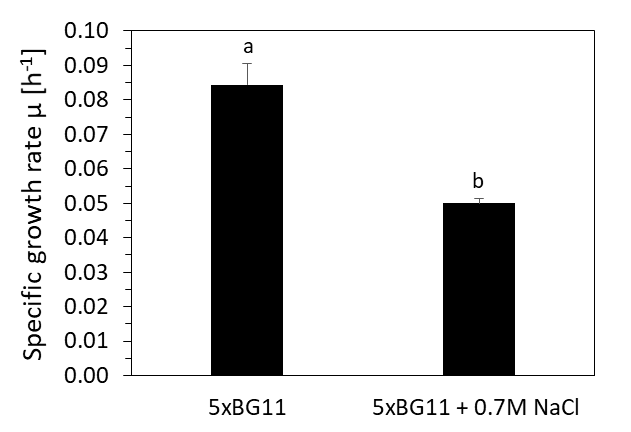
**

**Fig M** Specific growth rates of *Synechocystis* GT-L in the 5xBG11 cultivation medium (in which each single nutrient was five times increased when compared to standard BG11 medium), with and without the presence of 0.7M NaCl. The data represent averages from at least 5 successive growth rates (as determined within a quasi-continuous cultivation) after the growth stabilization under both treatments. The cultivation conditions were set such as to saturate growth of *Synechocystis* GT-L from 95%, i.e. 220 µmol(photons) m^-2^ s^-1^, 32°C and input CO_2_ concentration of 5 000 ppm. Differences in specific growth rates with the presence of NaCl are marked by letters above the particular columns (Tukey’s HSD post-hoc test following one-way ANOVA: p<0.05).

**Table A** Parameters of the photosynthesis-irradiance curves (P-I curves) of *Synechocystis* substrains GT-L, GT-B and PCC-B as shown in Figure 3. Data points from the P-I curves were fitted according to Platt et al. (1980) [1]. All experimental details are described in legend of Figure 3. α – photosynthetic efficiency as identified from the linear (light limited) part of the P-I curve, P max – maximal photosynthetic capacity.

|  | Red light |  | 25 µmol(photons) m^-2^ s^-1^ | | |  | 220 µmol(photons) m^-2^ s^-1^ | | |  | 660 µmol(photons) m^-2^ s^-1^ | | |  | 72 µmol(photons) m^-2^ s^-1^ | | |  |
| --- | --- | --- | --- | --- | --- | --- | --- | --- | --- | --- | --- | --- | --- | --- | --- | --- | --- | --- |
|  | Blue light |  | 25 µmol(photons) m^-2^ s^-1^ | | |  | 25 µmol(photons) m^-2^ s^-1^ | | |  | 25 µmol(photons) m^-2^ s^-1^ | | |  | - | | |  |
|  | White light |  | - | | |  | - | | |  | - | | |  | 526 µmol(photons) m^-2^ s^-1^ | | |  |
|  |  |  | GT-L | PCC-B | GT-B |  | GT-L | PCC-B | GT-B |  | GT-L | PCC-B | GT-B |  | GT-L | PCC-B | GT-B |  |
|  |  |  |  |  |  |  |  |  |  |  |  |  |  |  |  |  |  |  |
|  | α |  | 0.0010 | 0.0014 | 0.0016 |  | 0.0015 | 0.0017 | 0.0017 |  | 0.0008 | 0.0004 | 0.0004 |  | 0.0016 | 0.0009 | 0.0013 |  |
|  | P_max_ |  | 0.1093 | 0.1375 | 0.0892 |  | 0.2074 | 0.2277 | 0.1430 |  | 0.2391 | 0.2120 | 0.1812 |  | 0.2548 | 0.1951 | 0.1430 |  |
|  |  |  |  |  |  |  |  |  |  |  |  |  |  |  |  |  |  |  |

**Table B** Composition of fatty acids in the *Synechocystis* substrains PCC-B, GT-L and GT-B under 32 °C and 23 °C. The cultures were cultivated in a quasi-continuous regime under 220 µmol(photons) m^-2^ s^-1^ of red light, complemented with 25 µmol(photons) m^-2^ s^-1^ of blue light and at CO_2_ concentration in input gas 5 000 ppm. The values represent averages of at least three biological replicates, relative standard errors were lower than 5% in all cases and are not shown. The “t” represent trace amounts (< 1%). Variation in the lipids composition among the *Synechocystis* substrains was statistically not significant (Tukey’s HSD post-hoc test following one-way ANOVA: p>0.05).

| Fatty acid | | |  | 32°C | | | | |  | 23°C | | | | |  |
| --- | --- | --- | --- | --- | --- | --- | --- | --- | --- | --- | --- | --- | --- | --- | --- |
|  |  |  |  | PCC-B |  | GT-L |  | GT-B |  | PCC-B |  | GT-L |  | GT-B |  |
|  |  |  |  |  |  |  |  |  | mol % | |  |  |  |  |  |
|  | 14:0 |  |  | t |  | t |  | t |  | t |  | t |  | t |  |
|  | 16:0 |  |  | 56 |  | 54 |  | 54 |  | 52 |  | 52 |  | 53 |  |
|  | 16:1 |  |  | 1 |  | 2 |  | 2 |  | 2 |  | 3 |  | 3 |  |
| 14-methyl | 17:0 |  |  | t |  | t |  | t |  | t |  | t |  | t |  |
|  | 17:0 |  |  | t |  | t |  | t |  | t |  | t |  | t |  |
|  | 18:0 |  |  | 5 |  | 4 |  | 4 |  | 3 |  | 2 |  | 3 |  |
|  | 18:1 |  |  | 2 |  | 3 |  | 2 |  | 1 |  | 2 |  | 1 |  |
| Trans | 18:1 |  |  | t |  | t |  | t |  | 1 |  | t |  | t |  |
|  | 18:2 |  |  | 16 |  | 15 |  | 16 |  | 8 |  | 6 |  | 6 |  |
| α- | 18:3 |  |  | 2 |  | 3 |  | 2 |  | 10 |  | 11 |  | 10 |  |
| γ- | 18:3 |  |  | 17 |  | 18 |  | 18 |  | 17 |  | 16 |  | 17 |  |
|  | 18:4 |  |  | t |  | t |  | t |  | 6 |  | 8 |  | 7 |  |

**Analytical methods**

Composition of cellular fatty acids was estimated by modified method of [3]. Briefly, for each sample 100 ml of culture was centrifuged (4 000 g, 10 min), pellet was re-suspended in 10 ml of double deionized water and transferred to 15 ml falcon tube. The samples were centrifuged again, water was discarded and supernatants were re-suspended in 10 ml of hot isopropyl alcohol (60°C) containing ionol at 0.002%. The tubes were incubated in water bath at 65°C for 10 min, cooled down at laboratory temperature and stored at -20°C until further processing (up to 2 months). Before analysis, the samples were dried under nitrogen flow and the fatty acids were extracted in 1.5 ml of chloroform : methanol solution (2 : 1) at 60°C for 30 min. The extraction was repeated for two times, in case of insufficient phase separation 1 ml of 0.88% potassium chloride was added. After collecting chloroform phases, the extracts (with addition of internal standard - nonadecanoic acid, 19:0) were dried under nitrogen flow. Fatty acid methyl esters were prepared by transesterification in 1 ml of 3N methanolic HCl at 60°C for 90 min. After derivatization, the samples were extracted three times with 2.5 ml of n-hexane and dried under nitrogen flow. Finally, the extracts were dissolved in 1ml of n-hexane and injected into GC-MS for analysis. The analysis of fatty acids methyl esters was performed with TSQ Quantum XLS triple Quadrupole (Thermo Fisher Scientific, Waltham, MA, USA) on a 30 m, 0.25 mm (I.D), 0.25 μm capillary column (ZB-5MS) by injection of 1μl of sample in a splitless mode. The inlet pressure of carrier gas (helium) was 100 kPa at the initial oven temperature and helium flow rate was 1 ml min^-1^. The injection temperature was 250°C. The column temperature was increasing between 100 – 150°C in steps of 10°C min^-1^, and between 150 – 260°C in steps of 2.5°C min^-1^. The interface temperature was maintained at 250°C. The fatty acids were identified within the library built by standards measurements.

**References**:

1. Platt T, Gallegos CL, Harrison WG. Photoinhibition of photosynthesis in natural assemblages of marine phytoplankton. J Mar Res. 1980;38: 687–701.

2. Touloupakis E, Cicchi B, Torzillo G. A bioenergetic assessment of photosynthetic growth of Synechocystis sp. PCC 6803 in continuous cultures. Biotechnol Biofuels. BioMed Central; 2015;8: 133. doi:10.1186/s13068-015-0319-7

3. Sarsekeyeva FK, Usserbaeva AA, Zayadan BK, Mironov KS, Sidorov RA, Kozlova AY, et al. Isolation and Characterization of a New Cyanobacterial Strain with a Unique Fatty Acid Composition. Adv Microbiol. 2014;4: 1033–1043. doi:10.4236/aim.2014.415114
